# Redundancy maintains microbial ecosystem functional potential despite taxonomic shifts between serpentine and nonserpentine soils

**DOI:** 10.1099/mgen.0.001835

**Published:** 2026-09-22

**Authors:** Alexandria N. Igwe, Michelle E. Afkhami

**Affiliations:** 1 Department of Biological Sciences, Virginia Tech 926 W. Campus Drive, Blacksburg, VA 24060, USA; 2 Department of Biology, University of Miami 1301 Memorial Drive, Coral Gables, Florida 33146, USA

**Keywords:** functional redundancy, serpentine soil, shotgun metagenomics, taxa-function decoupling

## Abstract

Environmental filtering and buffering are complementary processes responsible for stabilizing ecosystem services across landscapes. The extent to which either of these processes structures microbial composition and functions in extreme soil systems can be highly context-dependent, requiring site-specific examination to elucidate general principles of community function under stress. Serpentine soils have high amounts of heavy metals and magnesium and low levels of plant nutrients and exist in close proximity to nutrient-rich and plant-productive nonserpentine soils, making them an ideal system for evaluating selection and redundancy under stress. Combining biogeographical field surveys and shotgun metagenomic sequencing of microbiomes from 23 pairs of serpentine and nonserpentine soils across California, we investigated selection and functional redundancy between disparate soil types. We hypothesized that the strong selective pressure present in serpentine soils would result in distinct bacterial and functional profiles. Bacterial and fungal community taxonomic compositions were indeed distinct between serpentine and nonserpentine soils, while archaeal communities were similar between soil types. In contrast, functional repertoires of all microbial community groups were largely similar between soil types, with many of the same taxa carrying out functions across soil types. Still specialized functions in serpentine soils represented adaptations to stress in contrast to the carbon-rich environment of nonserpentine soils. For example, specialized functions – such as siderophore biosynthesis proteins, which are involved in the biosynthesis of iron-chelating compounds – were distinctive features of serpentine soils and specialized functions notably had similar functional redundancy but distinct taxa carrying out the functions across soil types. These results highlight taxa that perform the functions that have been selected for survival in an extreme soil ecosystem and the functions that are most at risk in the face of environmental disturbances.

## Data Summary

 Raw sequences are available at https://www.ncbi.nlm.nih.gov/bioproject/PRJNA912991 and individual accession numbers are available in Table S5_sraresults.

**Impact Statement**

Soil microbes drive key ecosystem processes, but how microbial communities function in extreme soil environments is still being elucidated. Serpentine soils strongly influence the survival of microbes because of their low nutrient availability and high concentrations of magnesium and heavy metals. In this study, we used shotgun metagenomics to compare microbial community composition and functional potential in serpentine and adjacent nonserpentine soils. Bacterial communities were significantly different between soil types; additionally, communities in nonserpentine soils were enriched in genes associated with general nutrient uptake and metabolic activity, suggesting that these communities were optimized for resource acquisition and growth. In contrast, serpentine soils showed enrichment of functions related to stress tolerance and specialized transport, reflecting adaptations to metal toxicity and nutrient limitation. Despite differences in taxonomic composition, many metabolic functions were shared across soil types, indicating a degree of functional redundancy within the bacterial community. These findings highlight how soil chemistry shapes microbial community structure while maintaining essential ecosystem functions. This study advances our understanding of microbial adaptation and resilience in extreme soil environments and contributes to broader efforts to predict microbial responses to environmental change.

## Introduction

Soil microbial communities are responsible for a range of ecosystem services including nutrient cycling and supporting plant growth [1]. They are vital for conserving ecosystems, especially those characterized as rare or which host a large number of endemic species [2, 3]. Serpentine soils are one such environment. This soil is characterized by the weathering of ultramafic rock and contains high concentrations of magnesium and heavy metals, low calcium availability and low essential plant nutrients [4]. Comprising 1% of soils in California but hosting 13% of endemic plant species, serpentine soil is a biodiversity hotspot since many plants have evolved to grow on this harsh soil type and nowhere else [5–7]. Nonserpentine soils exist near serpentine soils but do not suffer from the same edaphic conditions as their neighbours and have greater plant productivity [8]. The differences in soil chemical properties and plant productivity offer a natural context to explore environmental filtering of microbial communities. Previous research has shown that the microbial communities associated with either soil type are distinct [9, 10] and the functional profile of microbes associated with serpentine soil has been described [11]. Still, the extent to which microbial functions correspond to functional stability across habitat types remains a question.

Broadly, the resilience of an ecosystem relies on an intricate balance between environmental filtering and functional redundancy. In terrestrial systems, soil pH, moisture availability, nutrient availability and other physicochemical properties often select for microbial communities with traits particularly well-suited for survival and reproduction in that particular environment (local adaptation) [12–14]. However, microbial traits that are adapted to narrow niches and specialized metabolic functions make microbial communities vulnerable to drastically changing conditions [15]. Complementarily, the presence of diverse taxonomic groups that respond differently to environmental conditions insures the community against disturbances [16–18]. Redundancy is a feature that can protect against spontaneous perturbations or poly-extreme environmental conditions [19]. The degree to which multiple species within a community can perform similar functions, functional redundancy, contributes to the ability of the environment to buffer against disturbances [20]. This feature also contributes to taxa-function decoupling, where ecosystem functions remain stable despite taxonomic compositional shifts [21]. Although environmental filtering and trait-based theory suggest that extreme soil chemistries may select for distinct functional traits, functional redundancy could lessen the effect of filtering and result in similar functional profiles despite differences in taxonomic composition [19].

Serpentine soils provide an excellent system with which to explore environmental filtering and functional redundancy. Previously, extreme soils, like serpentine soils, have been studied to understand adaptive traits that allow organisms, especially plants, to survive but relatively little research has specifically investigated microbial traits. Some *Mesorhizobium* strains have gene clusters on their accessory genome that facilitate growth in high-nickel environments, and those genes have the potential to be shared among community members through processes like horizontal gene transfer [22, 23]. Even as these genome islands and mobile genetic elements allow specialized functions, most of the genome and functional potential is similar between isolates. Functions related to heavy metal efflux in serpentine soils were also interpreted by assessing 16S rRNA amplicon sequences [24]. These studies highlight the outcome of long-term stress on the evolution of microbial populations and communities. Another study highlighted the roles of environmental filtering and dispersal limitation on microbial community assembly. In serpentinizing aquifers in Northern California, pH imposed a strong selective pressure on microbial community composition while slow fluid movement in the subsurface contributed to dispersal limitations between closely spaced wells [25]. These findings highlight the potential for both environmental conditions and spatial processes to structure microbial communities in serpentinizing environments. Still, a comprehensive assessment of functions using shotgun metagenomics has not been done across this unique ecosystem, possibly omitting adaptive microbial traits beyond heavy metal efflux.

Combining biogeographical field surveys and shotgun metagenomic sequencing of bulk soil from 23 pairs of serpentine and nonserpentine soils across California, we aimed to identify taxonomic composition and functional features of each soil type. Based on the distinct soil characteristics of serpentine and nonserpentine soils and the process of environmental filtering, we hypothesized that microbial community compositions and microbial functional profiles would be distinct and reflect adaptations to their respective soil chemistries [2, 9, 26]. We predicted that functions related to stress tolerance, secondary metabolite production and metal transport would be enriched in serpentine soils, whereas nonserpentine soils would contain more functions related to nutrient acquisition and carbon metabolism. To test this hypothesis, we used shotgun metagenomics to characterize microbial communities from paired serpentine and nonserpentine soils. Then, we quantified taxonomic composition across bacteria, archaea and eukaryotes and assessed functional potential using eggNOG annotations. This approach allowed us to examine archaeal and eukaryotic communities alongside bacteria within the same soil samples, which provided a broader molecular characterization of microbial ecology in serpentine soils than previous targeted studies. Even when environmental filtering alters community composition, overlapping functional capabilities among taxa can maintain ecosystem-level processes. With this in mind, the Functional Redundancy Index was calculated to determine the extent to which functions overlapped between serpentine and nonserpentine soils. By integrating patterns of taxonomic divergence, functional potential and redundancy, this study provides information that compares and contrasts the microbial composition and function associated with serpentine and nonserpentine soils and the communities’ potential to maintain those functions.

## Methods

### Soil collection

Soils were collected from 23 pairs of serpentine and nonserpentine Californian field sites from November to December 2020 (Fig. S1, available in the online Supplementary Material). We collected from sites that were previously reported as serpentine soils through personal communications and the USGS Data Series 414. An ethanol-sterilized garden spade was used to excavate soils at an average depth of 12.5 cm every metre across a 5 m transect that represented the soil chemistry of the site. These aliquots of soil were mixed and placed into a sterile 18 oz Whirl-Pak bag to form the one composite soil sample that was collected per site. Paired nonserpentine sites were identified visually within 5 miles of the serpentine site and then confirmed using the USGS Data Series 414 to be nonserpentine. The same method was used to excavate the nonserpentine soils. Collected soils were combined, then placed into a sterile Whirl-Pak bag on ice and subsequently stored in a −80 °C freezer until DNA extraction.

### Sample processing and microbiome sequencing

Soils were removed from the freezer and sieved using a 2 mm mesh sieve to remove large rocks and debris. DNA was extracted using the DNeasy PowerSoil Pro Kit (QIAGEN, Hilden, Germany;(https://www.qiagen.com/in) following standard protocols, with a minor modification that included extracting DNA from the same sample three times successively to increase potential DNA yield [27]. Quality DNA was recovered from 42 samples (21 paired sites, excluding Porterville and San Luis Obispo). DNA was extracted from sterile Milli-Q water and used as negative controls. DNA samples (42 soil samples and 2 blanks) were sent to the Integrated Microbiome Resource at Dalhousie University for metagenomic sequencing on an Illumina NextSeq 550 (150+150 bp PE) at 2× depth (~8 M PE reads=16 M single reads and 2.4 Gb/sample) with a PhiX spike-in, which is often used as a positive control in Illumina sequencing [28, 29].

### Data processing

Quality filtering of sequence reads was performed using BBDuk v. 38.98 from the Joint Genome Institute. First, adapter and PhiX-derived sequences were removed using the following parameters: k=31, hdist=1 and ftm=5. Then sequences were trimmed with the following parameters: ktrim=r, k=23, mink=11, hdist=1, tbo=t, maq=10, qtrim=r and trimq=20. Finally, trimmed forward and reverse fasta files were merged using the BBMerge v. 38.98. No metagenome assembly was performed.

### Taxonomic assignments and analysis

Taxonomic classification of sample reads was performed by uploading the trimmed forward and reverse FASTQ files as paired-end reads to the Kaiju Web Server v. 1.9.2 [30]. The NCBI blast *nr*+euk database was used which is a non-redundant protein database that includes bacteria, archaea, viruses, fungi and microbial eukaryotes. This method uses protein-level classification and is more sensitive than nt-based methods. Taxonomic information was formatted into a phyloseq object. To identify possible contaminant taxa, the prevalence method in decontam v. 1.18.0 with a threshold of 0.5 was used to identify taxa with a higher prevalence in negative controls [31]. Contaminant taxa were removed, and the abundance, richness and diversity of bacteria, eukaryotes and archaea were analysed separately.

Differences in alpha diversity (Shannon and Simpson indices) were identified using linear mixed-effects models with soil chemistry as a fixed effect and site as a random effect to account for paired sampling locations. Shannon diversity was used to simultaneously capture effects on both taxonomic richness and evenness of microbial communities, and Simpson diversity was used to assess the contribution of dominant taxa [32–34].

### Functional assignments and analysis

A read-based approach was used to characterize the functional potential of samples from serpentine and nonserpentine soils. Functional assignments were generated from quality-filtered, overlapping paired-end reads that were merged using BBMerge v. 38.98. The resulting merged reads were mapped to the NCBI non-redundant protein database using DIAMOND. These merged reads represent individual paired-end sequencing fragments for which the forward and reverse reads overlapped and were combined; they were not generated through metagenomic assembly or contig reconstruction. MEGAN 6 was used to assign functional taxonomy in accordance with the eggNOG 5.0 database [35, 36]. The DIAMOND+MEGAN version that was used was megan-map-Jan2021.db. This database contains manually curated orthologous groups from various sources ‘including Gene Ontology (GO), Kyoto Encyclopedia of Genes and Genomes (KEGG) and placed in a functional category as introduced in Clusters of Orthologous Groups (COG), euKaryotic Orthologous Groups (KOG) and archaeal Clusters of Orthologous Genes (arCOG)’ [37]. The output from this analysis was exported using the read-to-count flag associated with the daa2info tool. National Center for Biotechnology Information (NCBI) taxonomy data associated with each read was also retained and used for functional redundancy analysis (RDA).

The read-to-count output from each sample was combined into a large data frame, aggregated by orthologous group (rows) and sample name (columns), transformed into a matrix, then used to create a phyloseq object. Based on the distribution of taxonomic reads, the phyloseq object was subsetted to only include bacteria, and analysis was performed on this subsetted dataset. Contaminant functions were identified and removed using the prevalence method associated with the decontam v. 1.18.0 package with a 0.5 threshold.

### Alpha and beta diversity metrics

Differences in alpha diversity (Shannon and Simpson indices) of microbial functions were identified using linear mixed-effects models with soil chemistry as a fixed effect and site as a random effect (to account for paired sampling locations). Shannon diversity was used to capture simultaneous effects on both richness and evenness of functions, while Simpson diversity assessed the contribution of dominant functions.

Beta diversity was assessed using Bray-Curtis dissimilarity, which quantifies compositional differences in taxonomic or functional abundances between communities [38]. For taxonomic analyses, Bray-Curtis dissimilarities were calculated from microbial community composition, whereas functional analyses were performed using eggNOG COG abundance profiles. Differences between serpentine and nonserpentine soils were evaluated using PERMANOVA stratified by site, with dissimilarity as the response variable and soil chemistry and site as predictors. Distance based RDA (db-RDA) was performed on Bray-Curtis dissimilarities with ordinations constrained by soil chemistry, and multivariate dispersion (distance to centroid) was calculated to assess within-group variability.

### Biogeographic patterns in taxonomic and functional composition

To evaluate potential biogeographic structuring, we tested for distance-decay relationships between geographic distance and Bray-Curtis community dissimilarity. We complemented this analysis with variance partitioning to estimate the relative contributions of soil chemistry and geographic location to taxonomic and functional variation. RDA was used to test the independent effects of soil chemistry and geographic location, whereas partial RDA was used to evaluate each factor after accounting for the effects of the other.

### randomForest classification and differential abundance analysis

A randomForest v. 4.7-1.1 classifier was used to evaluate whether functional gene profiles (eggNOG annotations) could predict soil chemistry and differential abundance analysis using DEseq2 v. 1.38.3 was performed to identify potential functions that were significantly different between serpentine and nonserpentine soils. That taxa contributing to functions that were considered both predictive and significantly different were visualized in a heatmap. For visualization purposes, taxa were grouped by hierarchical clustering based on their correlation profiles with the selected COG functional categories. For each taxon, the maximum absolute correlation with any COG category was calculated. Within each cluster, the taxon exhibiting the highest absolute correlation was selected as the representative taxon for the heatmap.

### Functional redundancy

The degree of functional redundancy was calculated within and between soil chemistries using a functional redundancy index (FRI) [39]. Briefly, within-community functional redundancy index (FRIa) calculations quantified the diversity of bacterial phyla encoding the same functions within the community based on Shannon index. FRIa is a measure of how evenly multiple taxa (designated with an S) contribute to the same function within a community; higher values indicate more even contributions across taxa in both serpentine and nonserpentine soils. Between-community calculations quantified differences in the identity of taxa encoding the same metabolic function between communities based on Bray-Curtis dissimilarity. Between-community functional redundancy (FRIb) signifies to what extent the same or different taxa carry out the same functions across different communities with values ranging between 0 and 1. Higher FRIb values suggest that different taxa carry out the same functions between communities, whereas lower FRIb values indicate that the same taxa carry out the same functions between communities. To assess the relationship between functional redundancy and changes in gene abundance, Pearson’s correlation was performed using the log2Change results from DEseq2 and the difference between FRIa between soil types.

To identify potential functions with drastic differences in functional redundancy both between and within soil types, a list of functions exhibiting a large difference in FRIa between serpentine and nonserpentine soils was identified (*n*=565). To focus on robust ecosystem functions rather than taxon-specific artefacts, the set was further filtered to retain only functions with measurable redundancy across phyla (FRIb >1). This filtering yielded 39 functions that exhibited large differences in redundancy between soil types while being distributed across diverse taxonomic groups, making them suitable for evaluating community-level patterns of functional redundancy.

## Results

### Overview

The resulting libraries produced a total of 496.16 million reads with an average of 11.28 million reads per sample. The raw sequence reads generated for this project are available at GenBank under BioProject accession no. PRJNA912991. After quality control for taxonomic assignments, ~482.07 million reads were retained with an average of 10.96 million reads per sample. After removing contaminant taxa, negative controls and a sample that lacked adequate sequences (Trimmer-Serpentine), 41,326 taxa from 41 sites were identified. Of the total taxa, 36,752 belonged to the Kingdom Bacteria, 2,881 belonged to the Kingdom Eukaryota and 1,693 belonged to the Kingdom Archaea. For functional assignments and after removing contaminant functions, negative controls and a sample that lacked adequate sequencing (Trimmer-Serpentine), 2,171 functions from 37 sites were identified. Of the three major eggNOG categories, 1,099 functions were related to metabolism, 590 were related to cellular processes and signalling and 481 were related to information storage and processing.

### Taxonomic dominance differs between soil types despite similar richness and evenness

**Fig. 1.**
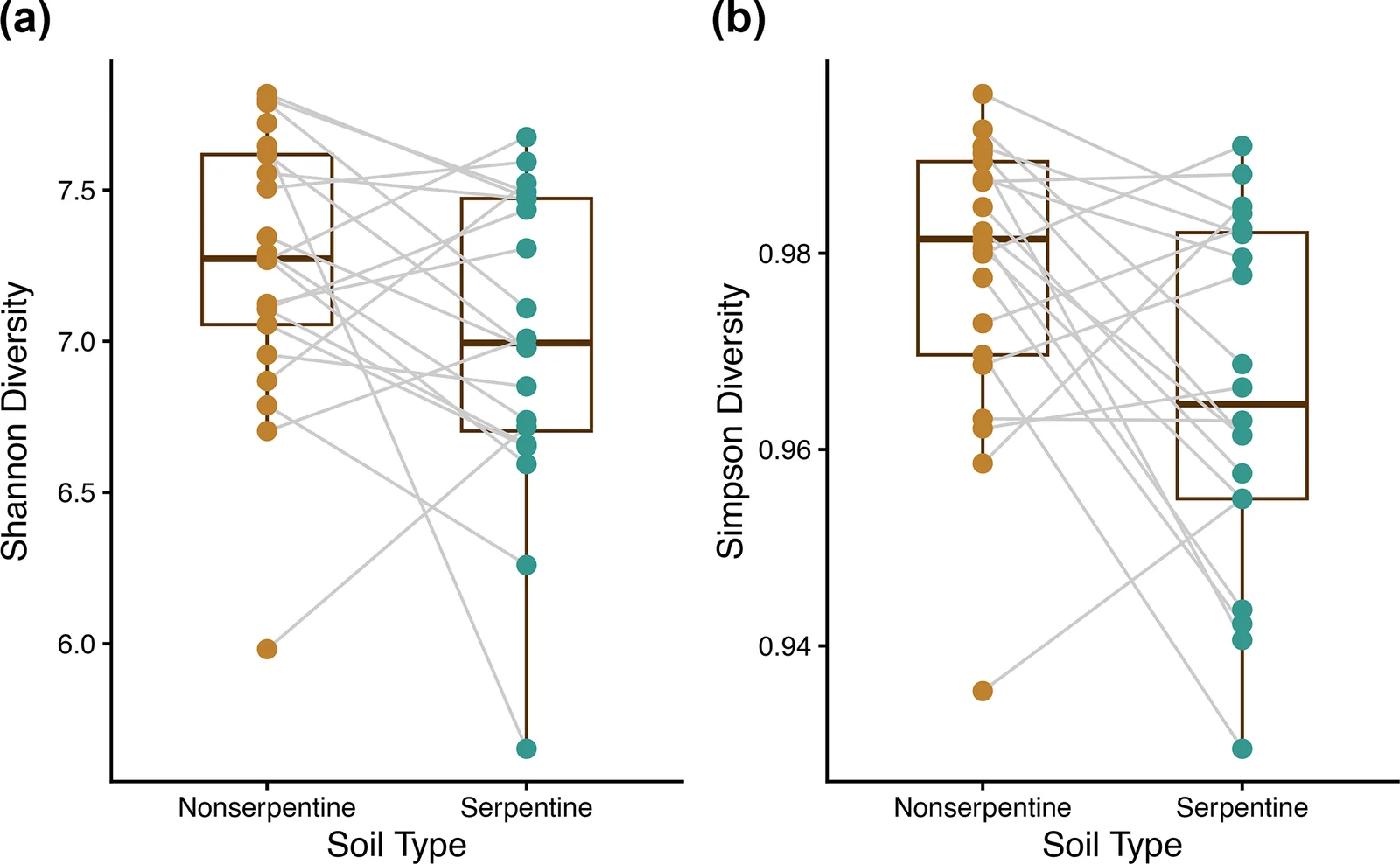
Alpha diversity of bacterial communities in serpentine and nonserpentine soils. Each point represents a sampling site, the bold horizontal line indicates the median and grey lines connect site pairs. Alpha diversity was overall lower in serpentine soils, and that difference was not significant using (a) Shannon (*β*=−0.240±0.135, *t*=−1.78, df=19.6, *P*=0.090), but significant using (b) Simpson diversity (*β*=−0.012±0.005, *t*=−2.62, df=19.8, *P*=0.017).

For bacteria, *Proteobacteria* dominated both soil types followed by *Actinobacteria* and *Acidobacteria*. In nonserpentine soils, *Candidatus Dormibacteraeota* were also present (Fig. S2). Alpha diversity (Fig. 1) was lower in serpentine soils relative to nonserpentine soils, but the effect was non-significant based on Shannon diversity (*β*=−0.240±0.135, *t*=−1.78, df=19.6, *P*=0.090) and significantly different based on Simpson diversity (*β*=−0.012±0.005, *t*=−2.62, df=19.8, *P*=0.017). This suggests that serpentine communities are dominated by a few taxa while overall richness and evenness remain comparable.

**Fig. 2.**
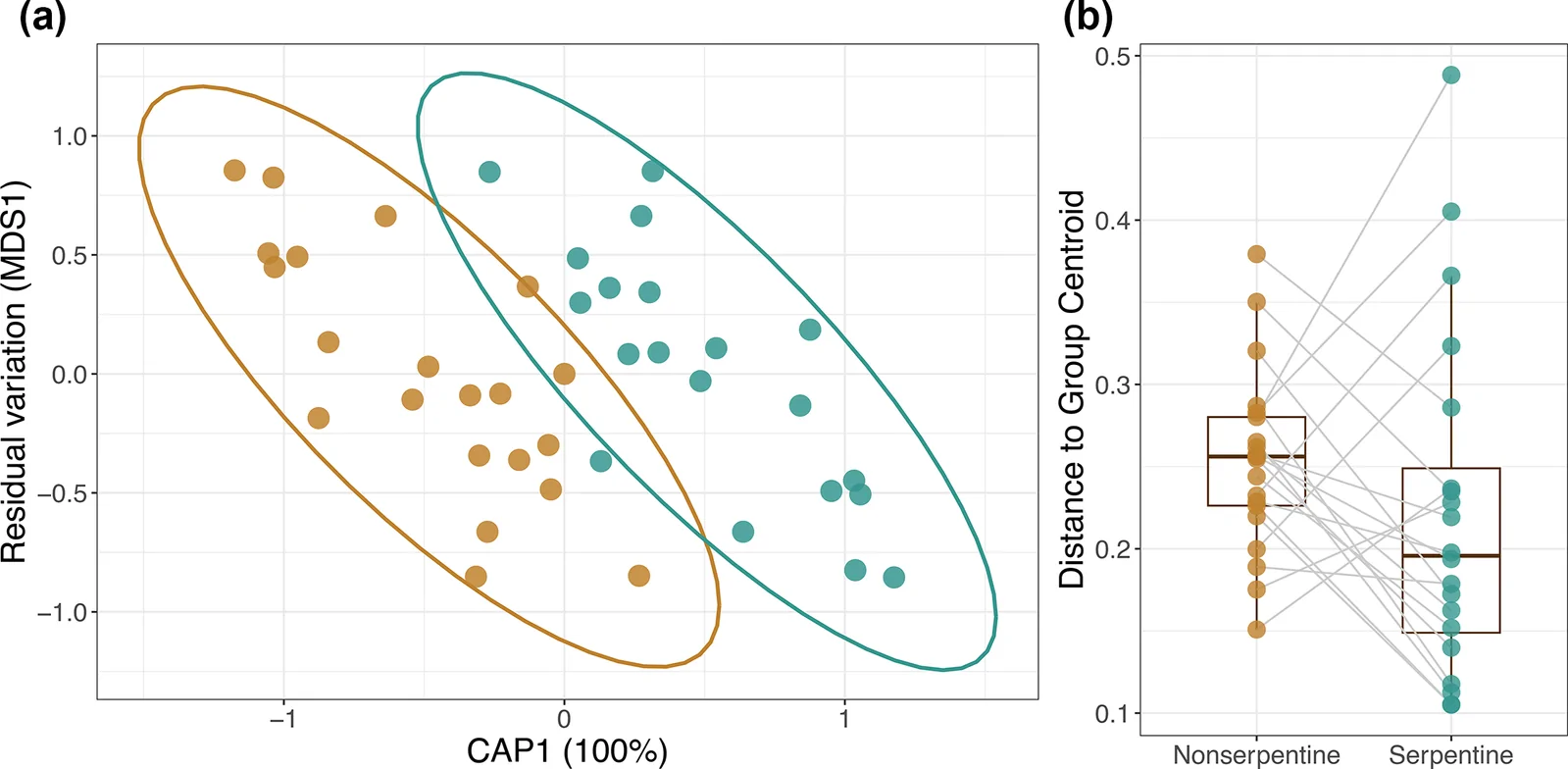
Db-RDA (CAP) of Bray-Curtis dissimilarity of serpentine and nonserpentine soil bacterial communities. Each point represents a sampling site, coloured by soil chemistry. (a) The x-axis (CAP1) shows variation constrained by soil chemistry, and the y-axis (MDS1) shows residual, unconstrained variation. Panel (b) shows the distance of each sample to its group’s centroids (lmer: *β*=−0.032±0.025, *t*=−1.29, df=20.2, *P*=0.211). Bacterial communities were significantly different between serpentine and nonserpentine soils (PERMANOVA: F_(1,19)_=3.045, *P*=0.003, *R*²=0.061).

Bray-Curtis dissimilarity of bacterial communities differed significantly between serpentine and nonserpentine soils within paired sites (Fig. 2; F_(1,19)_=3.05, *P*=0.004, *R*²=0.061), while variation among site pairs explained the majority of compositional differences (F_(20,19)_=1.40, *P*=0.004, *R*²=0.559). Distance based redundancy analysis (Db-RDA) constrained by soil chemistry shows separation of bacterial communities along CAP1 (constrained variation) and MDS1 (residual variation). CAP1 captured 100% of the variation constrained by soil chemistry, which represents 6.1% of total variation in bacterial community composition.

**Fig. 3.**
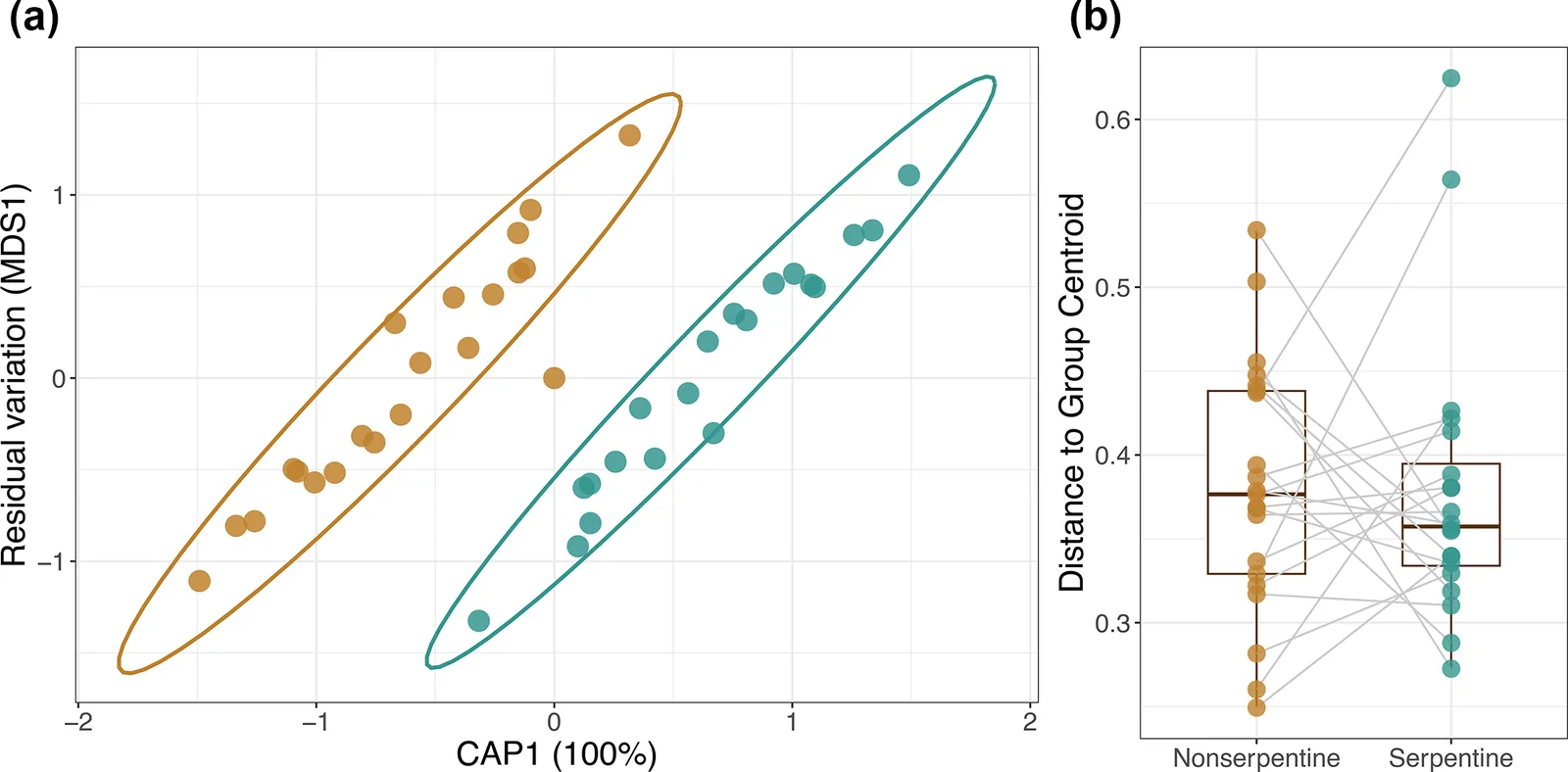
Db-RDA (CAP) of Bray-Curtis dissimilarity of serpentine and nonserpentine soil eukaryotic communities. Each point represents a sampling site, coloured by soil chemistry. (a) The x-axis (CAP1) shows variation constrained by soil chemistry, and the y-axis (MDS1) shows residual, unconstrained variation. Panel (b) shows the distance of samples to group centroids (lmer: *β*=−0.002±0.025, *t*=−0.08, df=39, *P*=0.937). Eukaryotic communities were significantly different between serpentine and nonserpentine soils (PERMANOVA: F_(1,19)_=5.39, *P*=0.001, *R*²=0.11).

Across both soil types, soil eukaryotic communities were dominated by Ascomycota with Basidiomycota being the next most prevalent phylum. Bacillariophyta and Rhodophyta were also common in serpentine soils, but made up less than 2% communities in nonserpentine soils (Fig. S3). Eukaryotic richness was not significantly different between serpentine and nonserpentine soils based on Shannon diversity (*β*=0.103±0.088, *t*=1.17, df=14.4, *P*=0.262) or Simpson diversity (*β*=0±0.002, *t*=−0.023, df=15.6, *P*=0.982) (Fig. S4). However, eukaryotic communities significantly diverged between serpentine and nonserpentine soils (Fig. 3 F_(1,19)_=5.39, *P*=0.001, *R*^2^=0.11) as well as by site pairs (F_(20,19)_=1.28, *P*=0.045, *R*^2^=0.511). Db-RDA constrained by soil chemistry shows separation of eukaryotic communities along CAP1 (constrained variation) and MDS1 (residual variation). CAP1 captured 100% of the variation constrained by soil chemistry, which represents 11% of total variation in eukaryotic community composition.

Unlike bacteria and eukaryotes, no differences were seen in the other prokaryotic lineage, archaea (Fig. S5). Euryarchaeota were the most abundant in both serpentine and nonserpentine soils followed by Thaumarchaeota. The species richness (Fig. S6) of archaea was not significantly different between serpentine and nonserpentine soils according to Shannon diversity (*β*=0.103±0.127, *t*=0.812, df=19.5, *P*=0.427) or Simpson diversity (*β*=0.001±0.001, *t*=1.28, df=39, *P*=0.210). Db-RDA constrained by soil chemistry shows apparent separation of archaeal communities by soil type. However, the archaeal communities between serpentine and nonserpentine soils (F_(1,19)_=1.69, *P*=0.107, *R*^2^=0.039) and sampling sites (F_(20,19)_=1.15, *P*=0.147, *R*^2^=0.526) were similar (Fig. S7). Likewise, we found similar degrees of variability in community composition among sites for all microbial groups. Specifically, using a linear mixed-effect model, we found the distance to the centroid for paired serpentine and nonserpentine soils was not significantly different in bacteria (*β*=−0.032±0.025, *t*=−1.29, df=20.2, *P*=0.211), archaea (*β*=−0.055±0.031, *t*=−1.77, df=39, *P*=0.084), and eukaryotes (*β*=−0.002±0.025, *t*=−0.08, df=39, *P*=0.937), indicating that differences in community composition were not due to differences in dispersion.

### Bacteria in nonserpentine soils weakly but significantly influenced by geographic distance

Distance-decay relationships were generally weak across microbial groups. Functional composition exhibited no evidence of distance-decay in either serpentine soils (Mantel *r*=−0.120, *P*=0.747) or nonserpentine soils (Mantel *r*=−0.007, *P*=0.309). Bacterial communities similarly showed little evidence of spatial turnover within serpentine soils (Mantel *r*=0.015, *P*=0.423). However, bacterial communities in nonserpentine soils exhibited a weak but significant positive relationship between geographic distance and community dissimilarity (Mantel *r*=0.208, *P*=0.040). Eukaryotic communities displayed weak positive but non-significant distance-decay relationships in both serpentine (Mantel *r*=0.126, *P*=0.195) and nonserpentine soils (Mantel *r*=0.136, *P*=0.137). Archaeal communities likewise exhibited no significant distance-decay, with weak relationships observed in serpentine soils (Mantel *r*=−0.003, *P*=0.450) and nonserpentine soils (Mantel *r*=0.074, *P*=0.252).

Variance partitioning revealed that soil chemistry and geography explained similar proportions of taxonomic variation in bacterial, eukaryotic and archaeal communities, whereas neither factor explained substantial variation in functional composition. Despite the generally weak distance-decay relationships, RDA revealed significant effects of geography and soil chemistry on microbial taxonomic composition. Soil chemistry significantly explained variation in bacterial (RDA: F_(1,39)_=3.00, *P*=0.005) and eukaryotic (F_(1,39)_=1.95, *P*=0.005) communities and remained significant after accounting for geographic location (Partial RDA: bacteria: F_(1,37)_=3.20, *P*=0.004; eukaryotes: F_(1,37)_=2.06, *P*=0.002). Consistent with the PERMANOVA results, archaeal communities exhibited substantially weaker responses to soil chemistry (RDA: F_(1,39)_=1.46, *P*=0.072) than bacterial and eukaryotic communities; although the effect became significant after accounting for geographic location (Partial RDA: F_(1,37)_=1.61, *P*=0.041). Geographic location significantly explained variation in bacterial (RDA: F_(2,38)_=1.90, *P*=0.011), eukaryotic (RDA: F_(2,38)_=1.73, *P*=0.001) and archaeal communities (RDA: F_(2,38_=1.96, *P*=0.004), and these effects remained significant after controlling for soil chemistry (Partial RDA: bacteria: F_(2,37)_=2.04, *P*=0.012; eukaryotes: F_(2,37)_=1.79, *P*=0.001; archaea: F_(2,37)_=2.03, *P*=0.001). In contrast, functional composition showed no significant effects of geography (RDA: F_(2,34)_=0.93, *P*=0.472) and only weak evidence of soil chemistry effects (RDA: F_(2,34)_=1.38, *P*=0.066).

### Specialized bacterial functions distinguish serpentine from nonserpentine soils despite overlap

**Fig. 4.**
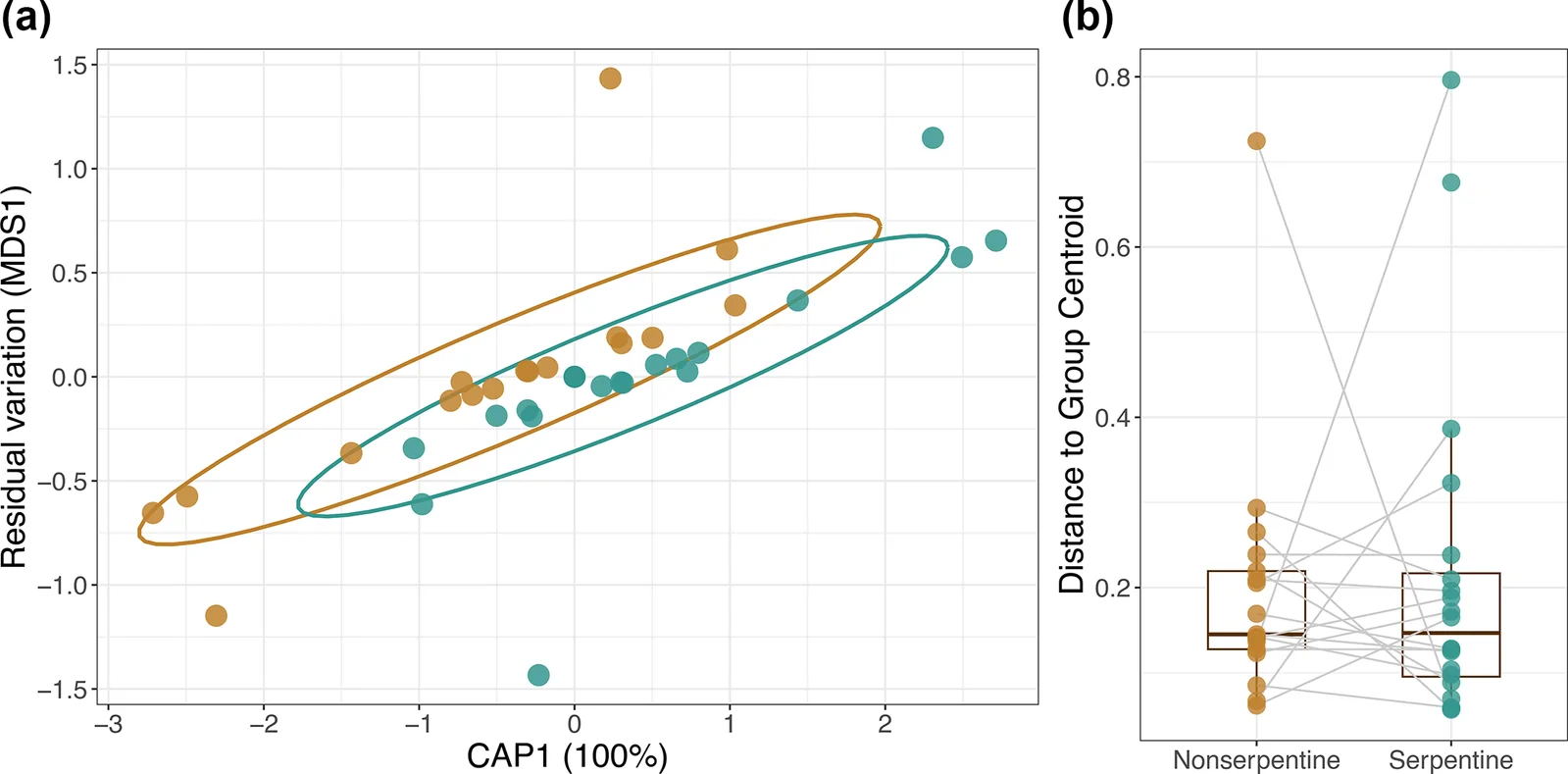
Db-RDA (CAP) of Bray-Curtis dissimilarity of serpentine and nonserpentine soil functional genetic repertoire. Each point represents a sampling site, coloured by soil chemistry. (a) The x-axis (CAP1) shows variation constrained by soil chemistry, and the y-axis (MDS1) shows residual, unconstrained variation. Panel (b) shows the distance of samples to group centroids (*β*=0.016±0.059, *t*=0.269, df=35, *P*=0.789). Functional potential was statistically similar between serpentine and nonserpentine soils (PERMANOVA: F_(1,16)_=1.044, *P*=0.391, *R*²=0.027).

In both serpentine and nonserpentine soils, the functional genetic repertoire had similar abundances (Fig. S8). The alpha diversity of functions between serpentine and nonserpentine soils (Shannon: F_(1,16)_=0.002, *P*=0.966; Simpson: F_(1,16)_=0.022, *P*=0.885) and sites (Shannon: F_(20,16)_=0.733, *P*=0.747; Simpson: F_(20,16)_=0.668, *P*=0.805) were not significantly different (Fig. S9). Soil chemistry (F_(1,16)_=1.044, *P*=0.391, *R*^2^=0.027) and sampling site (F_(19,16)_=1.126, *P*=0.710, *R*^2^=0.557) did not significantly influence functional genetic repertoires of the microbiomes (Fig. 4). This was the case even when outliers were removed, although there was a trend towards significance. Similarly, the variation in functional genetic composition was not significantly different between serpentine and nonserpentine soil (*β*=0.016±0.059, *t*=0.269, df=35, *P*=0.789).

**Fig. 5.**
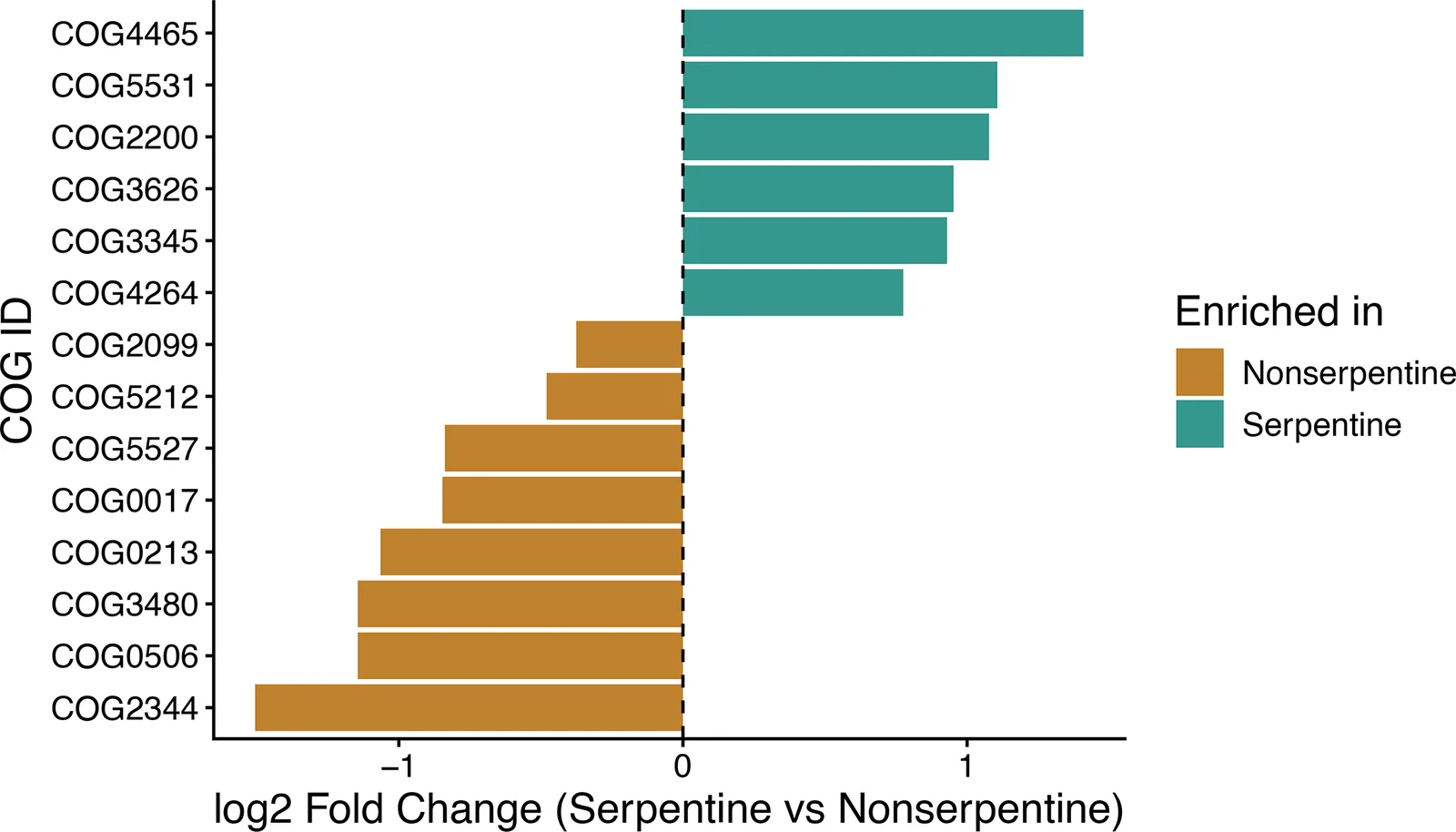
Bar graph depicting potential functions that are significantly different and predictive of soil chemistry. The log2 fold change is on the x-axis and COGs on the y-axis. Negative log changes are predictive of nonserpentine soils while positive log changes are predictive of serpentine soils. Description of COGs and statistical significance is listed in Table 1.

**Fig. 6.**
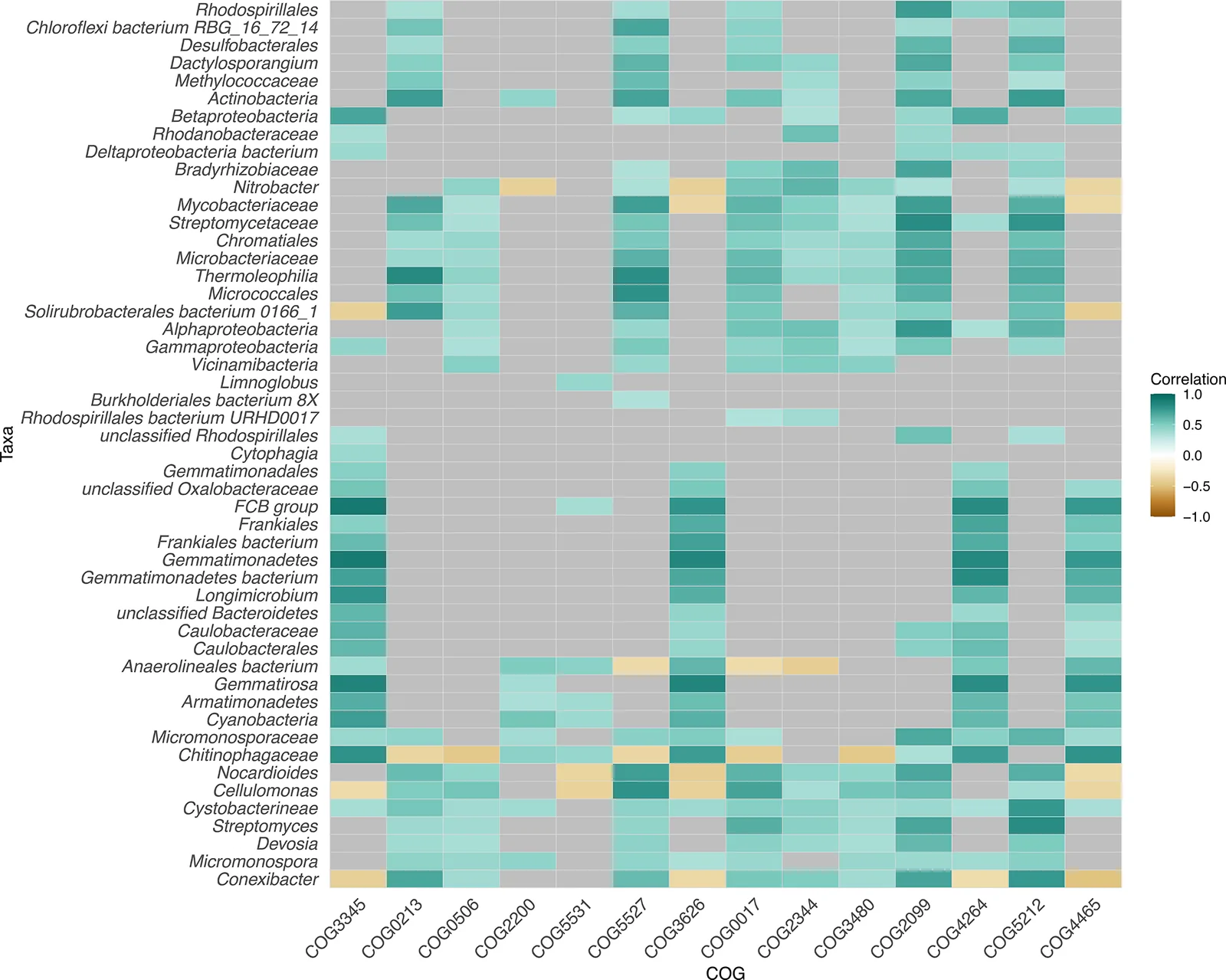
Heatmap depicting representative taxa that contribute to predictive and significantly different potential functions in nonserpentine and serpentine soils. Unlike previous figures where colours distinguish soil types, the colour scale in this heatmap represents correlation coefficients between taxa and functional categories. Grey panels signify the absence of the taxon–COG pair. Taxa displayed in the heatmap were selected based on their strongest associations with the focal COGs (listed in Table 1) and are not intended to represent taxa exclusive to either soil type.

While overall functional genetic repertoires were similar between soil types, we used randomForest models (randomForest 4.7–1.1) to evaluate whether differences in microbial functional gene profiles can successfully predict soil chemistry of sites and then used both the randomForest results and differential abundance analysis to determine which gene functions were important in differentiating serpentine and nonserpentine soils. PERMANOVA, randomForest and differential abundance analysis work at different resolutions, so that shifts in individual functions can be identified despite community-level similarities based on generalized housekeeping genes and processes. The randomForest model (ntree=15,001) achieved an out-of-bag error rate of 29.7%, corresponding to an overall classification accuracy of 70.3%. Classification success was higher for serpentine soils (85% correctly classified; class error=0.15) than for nonserpentine soils (53% correctly classified; class error=0.47), indicating that microbiomes from serpentine soils often house a specialized functional repertoire that is indicative of their soil chemistry. Further, differential abundance analysis was performed using DESeq2 (1.38.3) to identify gene functions that were significantly enriched in serpentine or nonserpentine soils. Functions that were considered to be both predictive via randomForest and significant via DESeq2 were visualized and evaluated further (Table 1, Fig. 5). The lowest taxonomic rank available was used when associating COGs with taxa, such that family, order, class, phylum or superphylum were used as the taxon label in the absence of genus assignment (Fig. 6).

**Table 1.** List of 14 COGs that were predictive of serpentine or nonserpentine soils and enriched in those same soils according to differential abundance analysis

COG ID	Description	Enrichment	log2 Fold Change	lfcSE	Stat	*P*_adj_
COG0213	The enzymes that catalyse the reversible phosphorolysis of pyrimidine nucleosides are involved in the degradation of these compounds and their utilization as carbon and energy sources or in the rescue of pyrimidine bases for nt synthesis (by similarity)	Nonserpentine	−1.064	0.284	−3.741	0.017
COG0506	Proline dehydrogenase	Nonserpentine	−1.144	0.332	−3.442	0.034
COG5527	Replication protein	Nonserpentine	−0.838	0.222	−3.773	0.016
COG0017	Asparaginyl-tRNA synthetase	Nonserpentine	−0.847	0.231	−3.657	0.022
COG2344	Modulates transcription in response to changes in cellular NADH NAD() redox state (by similarity)	Nonserpentine	−1.506	0.369	−4.084	0.008
COG3480	Domain protein	Nonserpentine	−1.144	0.332	−3.442	0.034
COG2099	Precorrin-6x reductase	Nonserpentine	−0.375	0.094	−3.978	0.010
COG5212	Phosphodiesterase	Nonserpentine	−0.480	0.111	−4.326	0.006
COG3345	α-Galactosidase	Serpentine	0.930	0.234	3.975	0.010
COG2200	Diguanylate cyclase phosphodiesterase	Serpentine	1.077	0.252	4.279	0.006
COG5531	SWI/SNF-related, matrix-associated, actin-dependent regulator of chromatin, subfamily d, member	Serpentine	1.107	0.308	3.593	0.024
COG3626	Phosphonate metabolism protein	Serpentine	0.952	0.191	4.991	0.001
COG4264	Siderophore biosynthesis protein	Serpentine	0.775	0.172	4.498	0.006
COG4465	DNA-binding protein that represses the expression of many genes that are induced as cells make the transition from rapid exponential growth to stationary phase; it is a GTP-binding protein that senses the intracellular GTP concentration as an indicator of nutritional limitations; at low GTP concentration, it no longer binds GTP and stops acting as a transcriptional repressor (by similarity)	Serpentine	1.410	0.345	4.082	0.008

Stat represents the test statistic (Wald z-statistic) used to calculate the p-value for each gene which is represented as the log2FoldChange divided by its standard error (lfcSE).

lfcSE, log2 fold change standard error.

In nonserpentine soils, the enriched COGs are primarily associated with core metabolic and cellular functions. These include pyrimidine nucleoside metabolism (COG0213), proline degradation (COG0506), replication and transcription processes (COG5527, COG0017, COG2344), general domain proteins (COG3480), precorrin-6x reductase activity (COG2099) and phosphodiesterase activity (COG5212). In contrast, serpentine soils are enriched in COGs linked to specialized metabolism, signalling and stress-adaptive functions, including sugar metabolism via alpha-galactosidase (COG3345), diguanylate cyclase/phosphodiesterase signalling (COG2200), chromatin remodelling (COG5531), phosphonate metabolism (COG3626), siderophore biosynthesis (COG4264) and transcriptional regulation in response to low GTP and stress (COG4465).

### Functional redundancy is not correlated with shifts in gene abundance

**Fig. 7.**
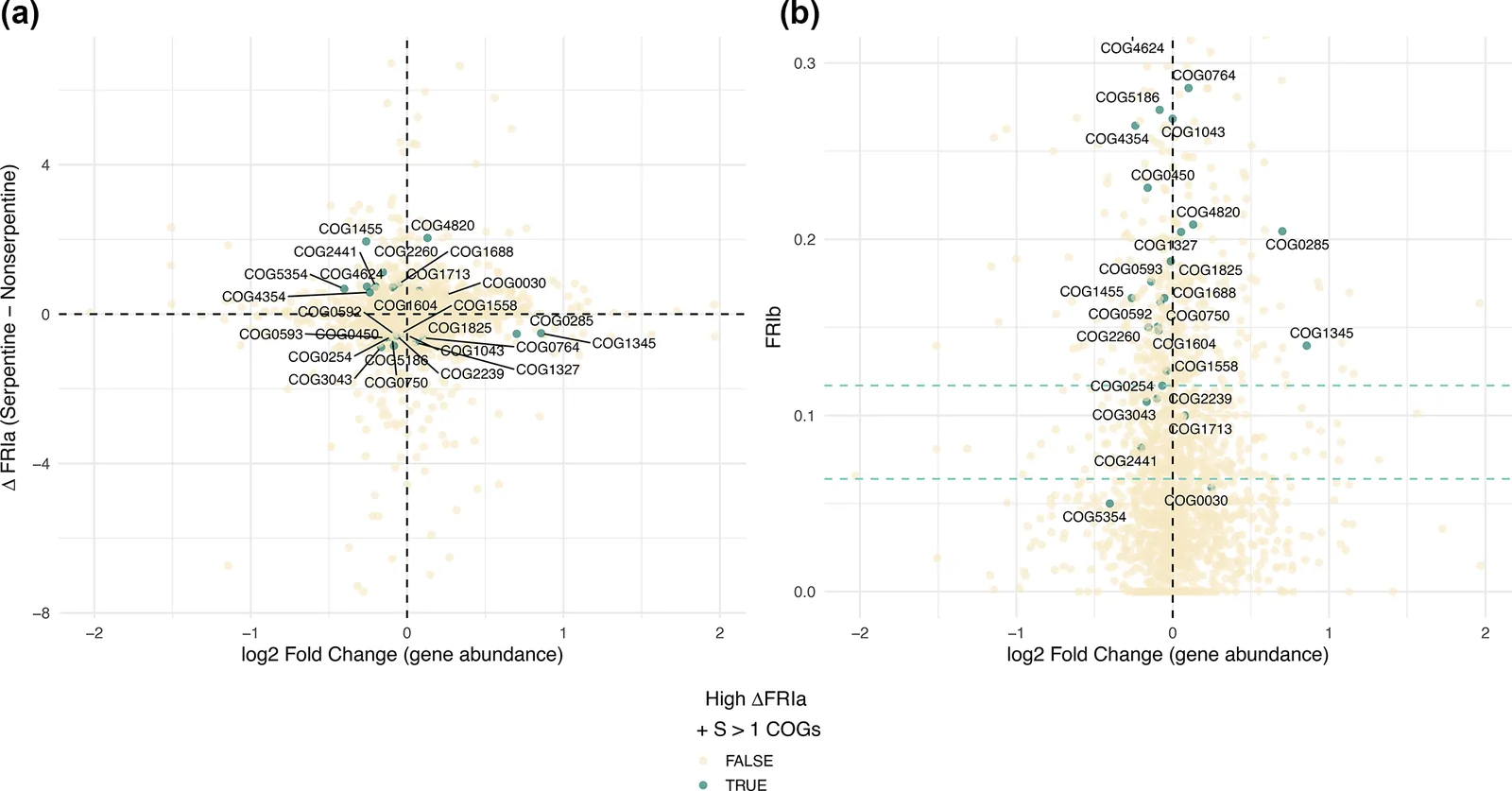
Correlation plots visualizing the relationship between changes in gene abundances and the difference between (a) within-community functional redundancy and (b) between-community functional redundancy. Labels represent the 39 functions that were shown to have large differences in within-community functional redundancies and were carried out by distinct phyla.

Given the conserved overall functional profile despite bacterial community taxonomic shifts, functional redundancy within and between serpentine and nonserpentine communities was explored (Fig. 7). FRIa of each COG was comparable between soil types, with redundancy of individual COGs ranging from 0 to 8.05 in serpentine soils and 0 to 7.96 in nonserpentine soil (Table S1). Across all functions, both soil types had an average FRIa of 3.42. The number of phyla contributing to any given function ranged between 1 and 7 in both soil types with an average of 5 for serpentine and 4 for nonserpentine. The average for FRIb was 0.11 with the lower 25% below 0.03, the median at 0.06 and the upper 25% above 0.12. Differences in FRIa were not correlated with gene abundance (Pearson *r*=0.02, *P*=0.37), indicating that shifts in taxonomic redundancy occur independently of changes in overall gene abundance. After filtering for functions that were associated with more than one phylum and had large differences in functional redundancy between serpentine and nonserpentine soils, 26 functions were identified (Table 2). These functions are performed by different phyla in each soil type and are more redundant in one soil type relative to the other. Like the larger set of functions, these are not correlated to shifts in gene abundance, indicating that changes in functional redundancy across serpentine and nonserpentine soils may be driven by shifts in specific taxonomic populations rather than shifts in gene abundances.

**Table 2.** Twenty-six COGs with high functional redundancy shifts (ΔFRIa) and high phyla differentiation (FRIb) between serpentine and nonserpentine soils. ‘S_Phyla_’ indicates the number of phyla performing each function; low S means few phyla contribute, whereas high S indicates broader redundancy

COG	Description	FRIb	S_Phyla_	FRIa nonserpentine	FRIa serpentine
COG5354	Component of the eukaryotic translation initiation factor 3 (eIF-3) complex, which is involved in protein synthesis and, together with other initiation factors, stimulates binding of mRNA and methionyl-tRNAi to the 40S ribosome (by similarity)	0.050	2	2.322	3.00
COG0030	Specifically dimethylates two adjacent adenosines (A1518 and A1519) in the loop of a conserved hairpin near the 3′-end of 16S rRNA in the 30S particle; may play a critical role in the biogenesis of 30S subunits (by similarity)	0.059	6	2.788	3.30
COG2441	Protein of unknown function (DUF1464)	0.082	2	3.907	4.65
COG1713	Metal-dependent phosphohydrolase	0.100	3	2.716	3.34
COG3043	Electron transfer subunit of the periplasmic nitrate reductase complex NapAB (by similarity)	0.108	2	5.477	4.59
COG0254	50S ribosomal protein L3-1	0.110	5	3.478	2.88
COG2239	Magnesium ion transmembrane transporter activity	0.117	5	3.675	3.10
COG1558	Flagellar basal-body rod protein Flgc	0.125	5	3.385	2.86
COG1345	Flagellar hook-associated	0.140	5	3.368	2.86
COG1604	CRISPR-associated RAMP protein, Cmr6 family	0.148	4	1.585	2.30
COG2260	rRNA processing	0.150	2	2.000	3.12
COG0750	Membrane-associated zinc metalloprotease	0.150	5	3.787	3.13
COG0592	DNA polymerase processivity factor activity	0.164	5	3.698	3.15
COG1455	PTS system	0.167	2	1.000	2.95
COG1688	CRISPR-associated protein Cas5	0.167	2	2.585	3.39
COG0593	It binds specifically dsDNA at a 9 bp consensus (dnaA box) 5′-TTATC CA A CA A-3′. DnaA binds to ATP and to acidic phospholipids (by similarity)	0.176	5	3.735	3.11
COG1825	This is one of the proteins that binds to the 5S RNA in the ribosome, where it forms part of the central protuberance (by similarity)	0.187	5	3.586	3.09
COG1327	Negatively regulates transcription of bacterial ribonucleotide reductase *nrd* genes and operons by binding to NrdR boxes (by similarity)	0.204	5	3.567	2.81
COG0285	Folylpolyglutamate synthase	0.205	5	3.692	3.16
COG4820	Ethanolamine utilization protein EutJ	0.208	2	1.585	3.63
COG0450	Alkyl hydroperoxide reductase	0.229	5	3.256	2.72
COG4354	Non-lysosomal glucosylceramidase that catalyses the conversion of glucosylceramide to free glucose and ceramide (by similarity)	0.265	3	3.451	4.03
COG1043	Involved in the biosynthesis of lipid A, a phosphorylated glycolipid that anchors the lipopolysaccharide to the outer membrane of the cell (by similarity)	0.268	5	3.424	2.86
COG5186	PolyA polymerase	0.273	2	4.620	3.77
COG0764	Involved in unsaturated fatty acids biosynthesis; catalyses the dehydration of short-chain β-hydroxyacyl-ACPs and long-chain saturated and unsaturated β-hydroxyacyl-ACPs (by similarity)	0.286	6	3.182	2.55
COG4624	Metallo-sulphur cluster assembly	0.333	2	1.585	2.32

## Discussion

### Differentiation in soil microbiome composition but not overall functional genetic capacity

This study hypothesized that the composition and functional profile of microbes associated with stressful serpentine soils would be distinct from those associated with nonserpentine soils due to edaphic selective pressures and environmental filtering. Retaining much of the characteristics of their parent material, serpentine soils have high amounts of heavy metals, low Ca:Mg ratios and limited availability of essential plant nutrients. These features provide a unique edaphic environment that can select for microbial taxa with specific physiological and metabolic traits that are adapted to metal stress and minimal nutrients. In fact, early cultivation-based methods showed that serpentine soils host distinct bacterial communities, hinting at the strong selective pressures present in serpentine soils [40]. In line with that study and using metagenomic approaches, our study found that serpentine and nonserpentine soils selected for dissimilar fungal and bacterial but not archaeal communities or functional profiles. Distance-decay analyses revealed generally weak relationships between geographic distance and community dissimilarity, with significant turnover detected only for bacterial communities in nonserpentine soils. In contrast, variance partitioning demonstrated significant geographic effects on bacterial, archaeal and eukaryotic taxonomic composition, even after accounting for soil chemistry. The effects of soil chemistry remained significant after accounting for geography, and geographic effects remained significant after accounting for soil chemistry, indicating that both environmental filtering and spatial processes contribute to microbial community assembly across California. Functional composition showed neither significant distance-decay nor significant geographic structure. These results indicate that microbial taxa exhibit spatial organization across California, whereas functional repertoires remain comparatively conserved despite environmental and geographic variation. Together, these results indicate that microbial taxa exhibit substantial spatial and environmental structuring, whereas functional repertoires remain comparatively conserved despite environmental and geographic variation. The significant contribution of soil chemistry to taxonomic composition supports the prediction that serpentine soils act as strong environmental filters. Previous work has shown that microbial community composition differs among serpentine habitats and responds to soil disturbance [10]. Our results extend these findings by demonstrating that soil chemistry structures microbial communities across broad geographic scales and multiple microbial domains while functional potential remains relatively stable. Furthermore, bacterial communities in either soil type maintained similar abundances of taxa, but communities from the high-stress serpentine soils were dominated by a few taxa while those from nonserpentine soils were more even. This result did not align with our hypothesis but instead reinforces the idea that both soil types have similar overall functional genetic repertoires that are carried out by distinct bacterial communities. Nonetheless, randomForest and differential abundance analysis revealed that despite overall function similarity between soil types, some functional capacities such as siderophore biosynthesis protein and α-galactosidase were enriched in serpentine soils, while proline dehydrogenase and precorrin-6x reductase were enriched in nonserpentine soils.

Studies have shown that bacterial communities can have distinct functional capacities across soil types [26, 41] and ecosystems [42]; still, there are others that show that functions are largely similar across the globe. In a study using 845 publicly available shotgun metagenomes collected distinctly across 17 climate zones, for example, it was found that functional potentials were more similar than microbial taxonomies and that climate and soil properties changed microbial taxonomies more than functions [43]. In a similar study which simulated biodiversity loss, it was found that functional potentials remained relatively stable despite significant changes in microbial taxonomies [44]. Generally, environmental conditions can alter the distribution of microbial traits and ecological strategies within communities [45], and our results suggest that serpentine soil chemistry primarily restructures microbial assemblages while maintaining substantial overlap in functional potential. This pattern is consistent with taxon-function decoupling, which serves as an adaptive strategy to protect against multiple disturbances [46, 47], since changes in taxonomic presence, absence or abundances do not significantly shift functions. One study showed that, while decoupling is a feature of some soils, many microbial functions are performed by a few dominant species [48], which is in line with the compositional and functional features associated with serpentine soils in this study.

### Differentiation of key functional genetic capacities and taxa responsible for functions between soil chemistries

Despite the similarities in overall functional repertoires of the microbiome, randomForest analysis identified specific functional genetic capacities that characterize nonserpentine soils, and to a greater extent, serpentine soils. Additionally, differential abundance analysis revealed functional gene groups that were significantly different between soil types. One of the 14 functions that were identified as important for differentiating among soils by both the randomForest models and the differential abundance analysis is siderophore biosynthesis protein (COG4264). Siderophores are a type of low-molecular-weight organic compounds that bind to iron. Over 20 plant-growth-promoting genera have been shown to produce these compounds and it co-occurs with other plant-growth-promoting properties like nitrogen fixation. The production of siderophores has been explored primarily in nutrient-poor and iron-limited environments, including in humans [49, 50]. Serpentine soils have the distinction of being nutrient-poor but iron-rich, suggesting that siderophore synthesis may play a different role in the ferromagnesian-based soil. Genes related to this function were highly correlated with the Fibrobacteres-Chlorobi-Bacteroidetes Group, *Frankiales*, *Gemmatirosa*/*Gemmatimonadetes* and *Chitinophagaceae* and were negatively correlated with *Conexibacter*. Siderophore biosynthesis was associated with two phyla in both nonserpentine and serpentine soils with comparable within-community functional redundancy. Based on between-community functional redundancy, the phyla that contribute to this function are similar in both soil types but phyla in serpentine soils contribute to the function more evenly than those in nonserpentine soils. Compared to serpentine soils, nonserpentine soils have significantly less iron, so siderophore biosynthesis may serve an expected role, but further research is needed to discern how siderophore biosynthesis contributes to ecosystem services in serpentine environments. Phosphonates (COG3626) are also important for nutrient acquisition and have been shown to be enriched in marine and terrestrial serpentinizing ecosystems [51]

Another interesting function identified as important was α-galactosidase (COG3345), which is part of the Raffinose Family Oligosaccharides that hydrolyse complex carbohydrates including α-galactoside-banded glycosides, such as stachyose, raffinose and verbascose [52]. It plays an important role in the carbon cycle by breaking down the complex sugars found in plant biomass releasing essential nutrients that support soil and plant health. The enzyme has been associated and isolated from thermophilic, biofilm-forming bacteria [53–55] and has economic value in processing agricultural waste [56]. In this study, the function was positively associated with *Gemmatirosa*, *Chitinophagaceae*, *Cyanobacteria* and *Longimicrobium*, to name a few. It was negatively correlated with *Cellulomonas* and *Conexibacter*. *Cellulomonas* is part of a genus whose defining characteristics is the degradation of cellulose [57]; the inverse relationship between this function and *Cellulomonas* suggests interesting niche overlap and competition between other populations in the community. This function was associated with six phyla in nonserpentine soils and four phyla in serpentine soils. Despite being represented across more phyla in nonserpentine soils, lower within-community functional redundancy suggests that a relatively small subset of those phyla contributed disproportionately to the function. In contrast, the function was distributed more evenly among the four associated phyla in serpentine soils.

Other functions related to biofilm formation were also identified such as diguanylate cyclase phosphodiesterase (COG2200), which plays a crucial role in the transition between planktonic and biofilm lifestyles [58]. Additionally, microbial biofilms improve plant growth by increasing the water-holding capacity of soil through improved soil aggregation [59]. In some microbes, the function impacts motility but does not induce biofilm formation. Instead, it regulates their virulence factors [60]. Virulence factors are responsible for many plant diseases and can also be shared within microbial communities, making previously beneficial microbes dangerous for plants [61, 62]. The role this function plays in serpentine soils remains to be explored; however, low plant productivity in serpentine soils imposes unique stressors on bacterial communities that may be alleviated by biofilm formation. This function was positively correlated with *Anaerolineales*, *Micromonospora*, *Cystobacterineae* and *Cyanobacteria* and negatively correlated with *Nitrobacter*. Given the fact that *Anaerolineales* is non-motile [63], alternative roles of this function cannot be ignored. In fact, this function has also been shown to facilitate secondary-metabolite production [64], which is in accordance with the positive correlation to *Micromonospora*, an important source of antibiotics [65]. This function is associated with four phyla in nonserpentine soils and five phyla in serpentine soils, with the function being dominated by a few of the phyla in serpentine soils and shared more evenly in nonserpentine soils. Overall, the populations performing this function are similar across soil types.

### Functional redundancy reveals important and vulnerable serpentine-associated functions

Although there is no correlation between changes in gene abundances and differences in functional redundancy between serpentine and nonserpentine soils, COGs that exhibit a positive log2 fold change and relatively large ΔFRIa represent COGs that are both enriched and highly redundant in the ecosystem. They may be particularly important for supporting the unique ecosystem services hosted on serpentine soils since their functions are carried out by many phyla. Some COGs, on the other hand, are more abundant in serpentine soils yet more redundant in nonserpentine soils; these COGs may be more vulnerable to large environmental changes in serpentine soils since their functions in serpentine soils depend on a few phyla. Twenty-six functions were identified to have large differences in redundancy and taxon roles across serpentine and nonserpentine soils. One of these, alkyl hydroperoxide reductase (COG0450) protects against oxidative stress, a major side-effect of UV radiation and other stressors [66]. In plants, folylpolyglutamate synthase (COG0285) has been shown to regulate nitrogen metabolism under nitrogen-limiting conditions [67] and metal-dependent phosphohydrolase (COG1713) has been shown to use many divalent metals to protect microbes against oxidative and heat stress [68, 69]. Other metal-dependent enzymes and transporters were identified, including metallo-sulphur cluster assembly (COG4624), membrane-associated zinc metalloprotease (COG0750) and magnesium ion transmembrane transporter activity (COG2239), which highlight adaptive functions that correlate to life on serpentine soils. The between-community functional redundancy of these functions ranges are relatively high, indicating that a few dominate taxa perform these functions, making them less buffered against environmental fluctuations compared to those that are less specialized.

## Conclusion

This study used historical physicochemical soil data to identify serpentine and nonserpentine soils. Replicate studies that increase sample size or extensively characterize soil properties will allow a better understanding of the correlation between soil chemistry and microbial structure and function. Still, the results of this study identify iron-binding, metal-transporting, biofilm-forming and antibiotic-producing functions as predictive and important features of serpentine soils. Furthermore, it identifies taxa that contribute to these functions across soil types and measures how redundant the function is within either soil type and the degree to which the same taxa carry out the function across soil types. Shifts in redundancy were shown to be distinct from changes in gene abundance and results suggest that changes in taxonomic diversity contribute to redundancy. These results have strong implications for conservation ecology, especially in a biodiversity hotspot like serpentine soil. Some functions identified here are enriched in serpentine soil but less redundant and are vulnerable to disturbances. Future culture-dependent experiments that characterize the physiological properties of these isolates or create synthetic communities may provide information on which taxa are coupled to which functions and how they work together to maintain bacterial communities and ecosystems.

Overall, this study emphasizes a precise relationship between environmental filtering and environmental buffering. Despite strong taxonomic divergence driven by serpentine geochemistry, the overall suite of functional genes remains stable across soil types, with some specialized functions acting as predictive features for serpentine and nonserpentine soils. Ultimately, understanding the balance between environmental filtering and functional redundancy will be critical for predicting how soil microbiomes respond to disturbance and sustain biogeochemical functions across diverse and extreme environments.

## Supplementary material

10.1099/mgen.0.001835 Supplementary Material 1.

10.1099/mgen.0.001835 Supplementary Material 2.

## Abbreviations

arCOG: archaeal Clusters of Orthologous Genes
COG: Clusters of Orthologous Groups
db-RDA: distance-based redundancy analysis
FRI: functional redundancy index
FRIa: within-community functional redundancy index
FRIb: between-community functional redundancy index
GO: Gene Ontology
KEGG: Kyoto Encyclopedia of Genes and Genomes
KOG: euKaryotic Orthologous Groups
NCBI: National Center for Biotechnology Information
RDA: redundancy analysis
