# Supplementary material for: Redundancy maintains microbial ecosystem functional potential despite taxonomic shifts between serpentine and nonserpentine soils
Source: Microb Genom. 2026 Sep 22;12(9):001835. doi: 10.1099/mgen.0.001835 (PMC13597068; doi:10.1099/mgen.0.001835)
Supplement: Supplementary file 1 [file mgen-12-01835-s001.pdf]

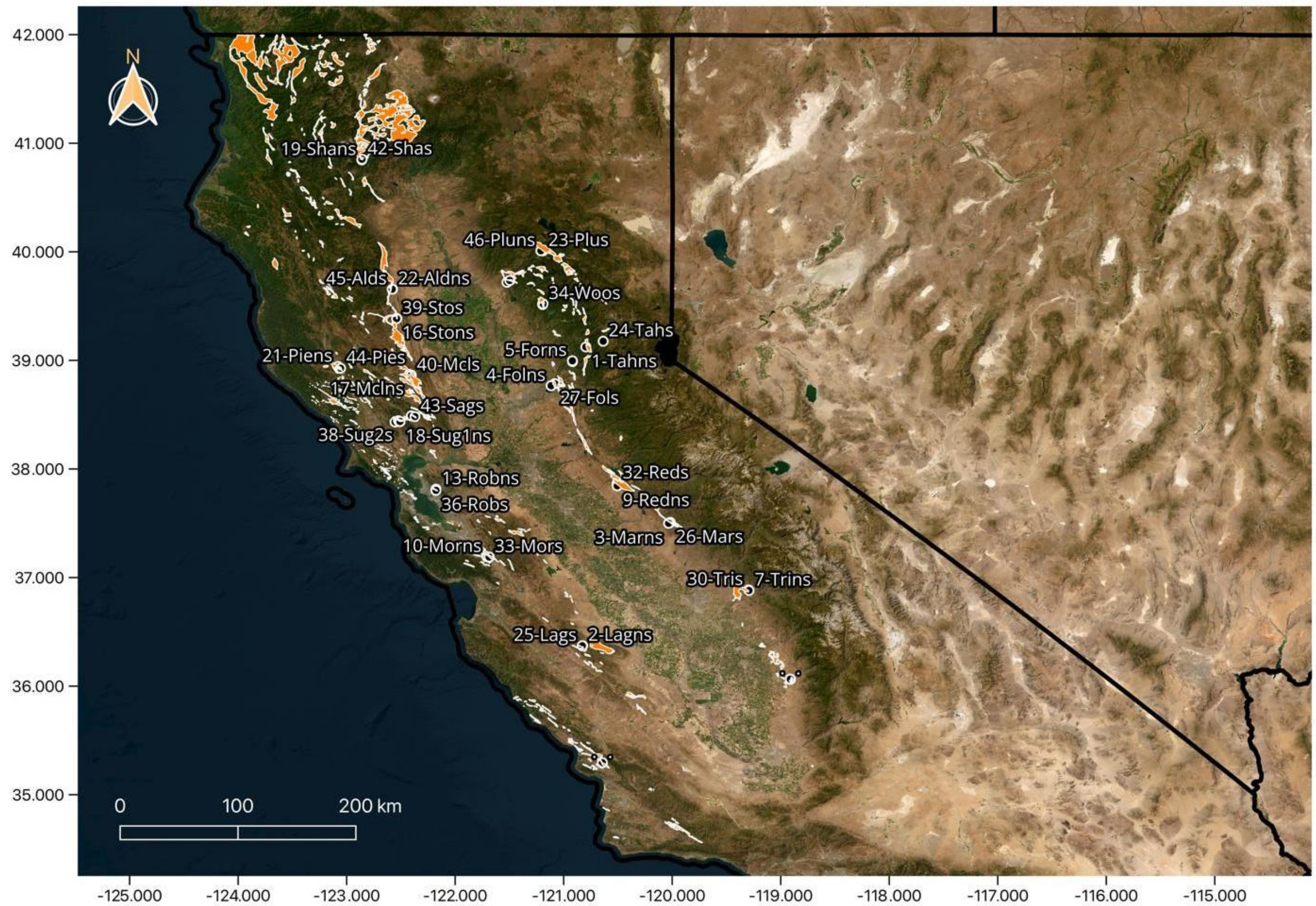

Supplemental Figure 1 - Map of sampling sites

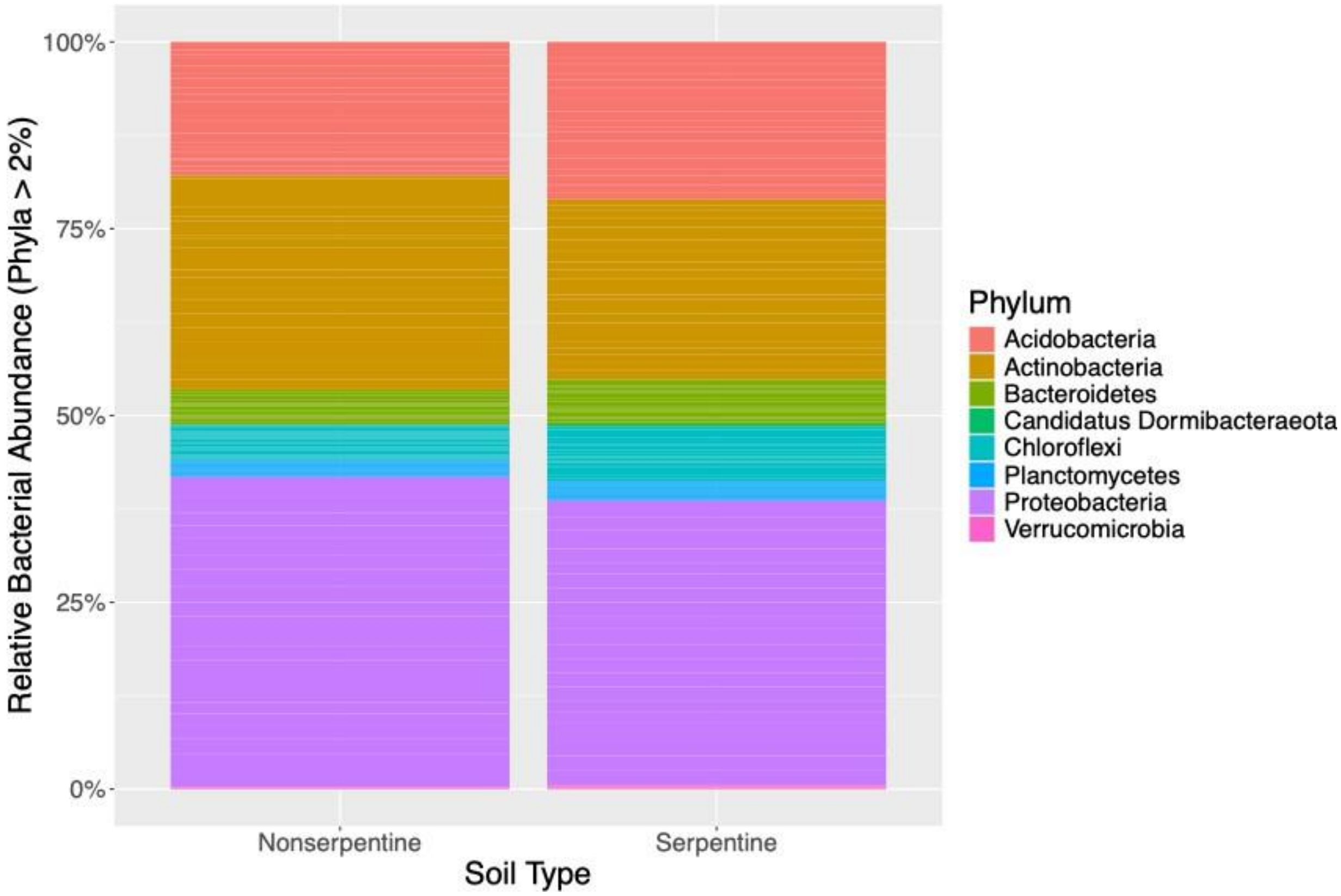

Supplemental Figure 2 - Bacterial community composition

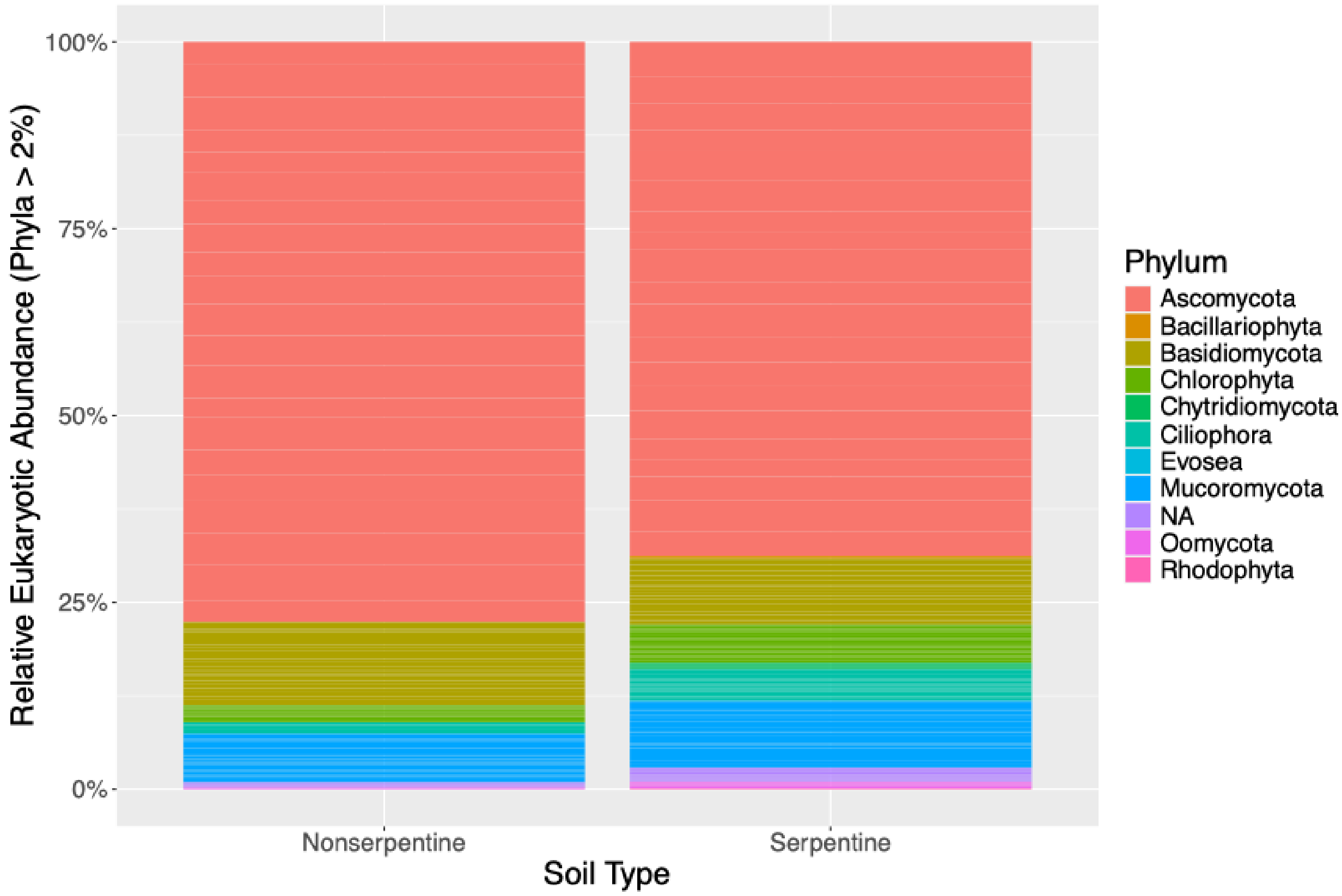

Supplemental Figure 3 - Eukaryotic community abundance

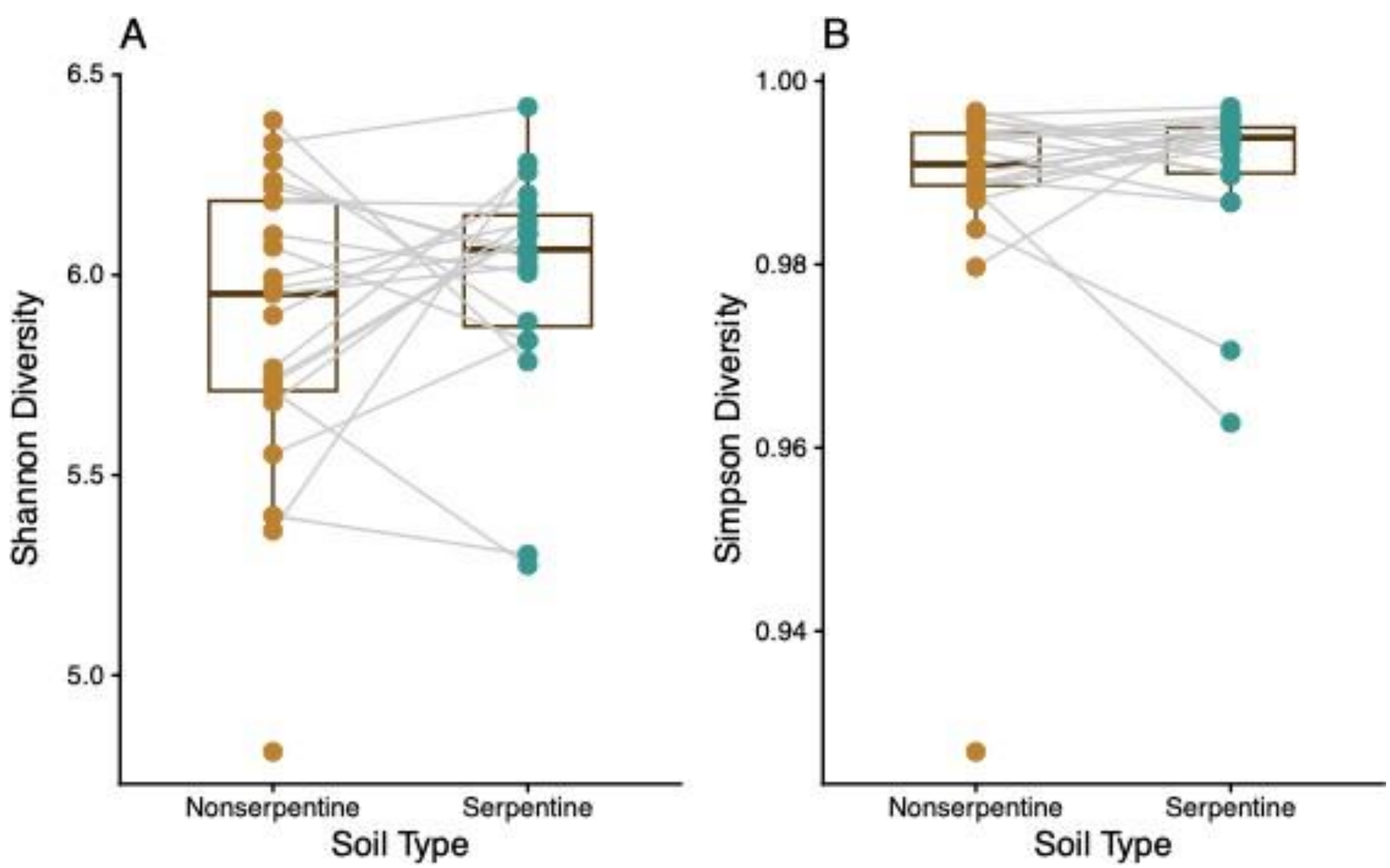

Supplemental Figure 4 - Eukaryotic community richness

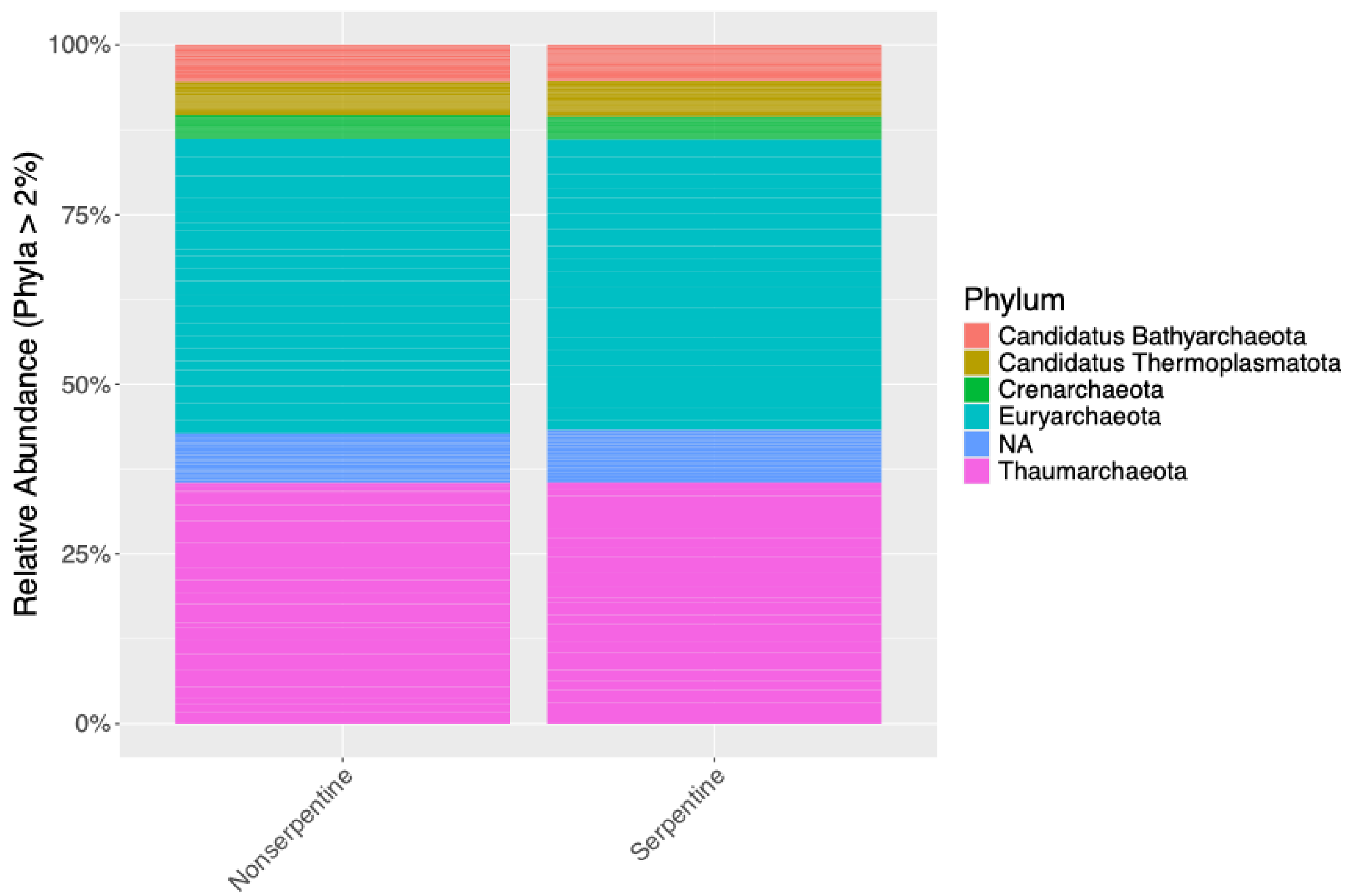

Supplemental Figure 5 - Archaeal community composition

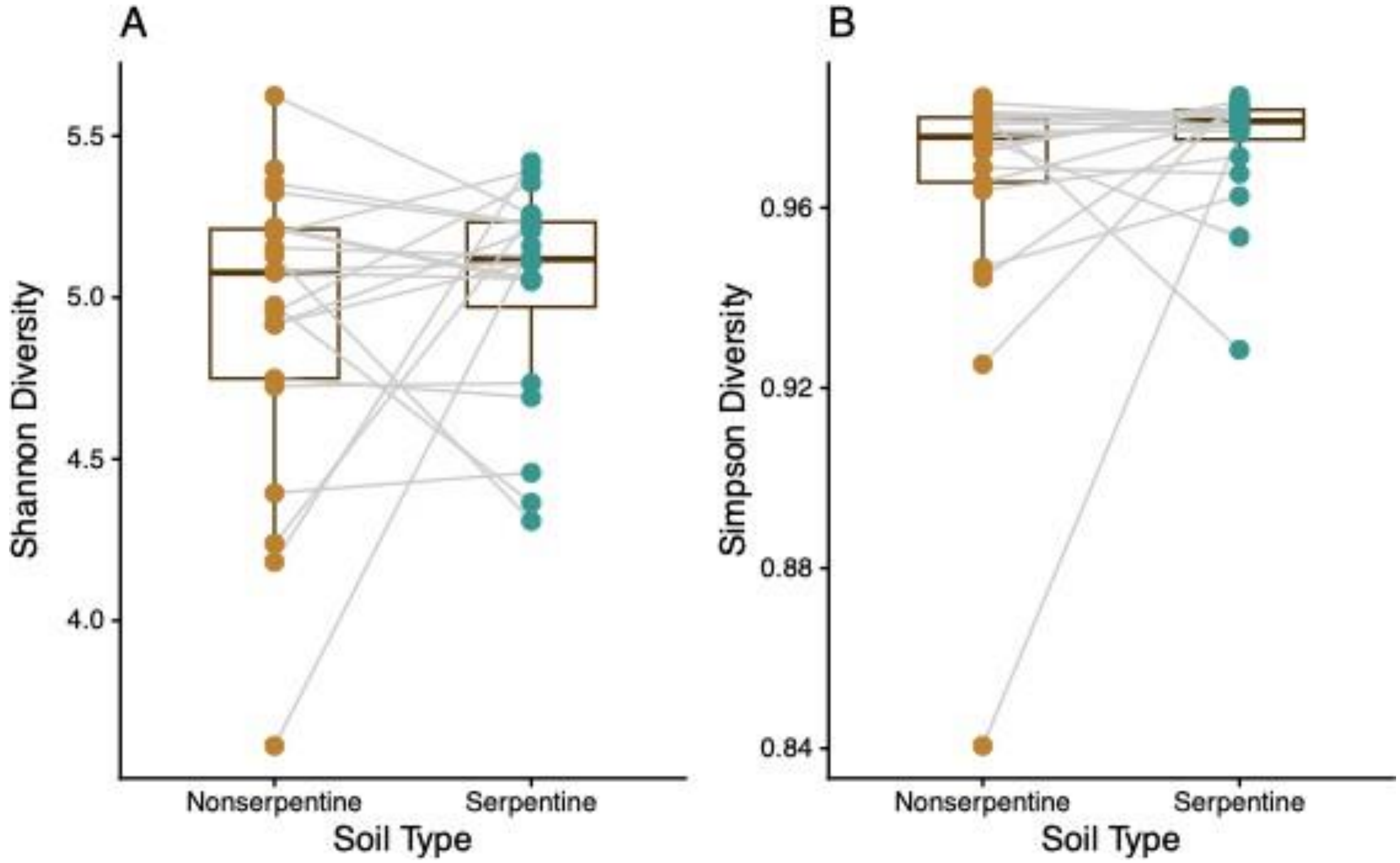

Supplemental Figure 6 - Archaeal community richness

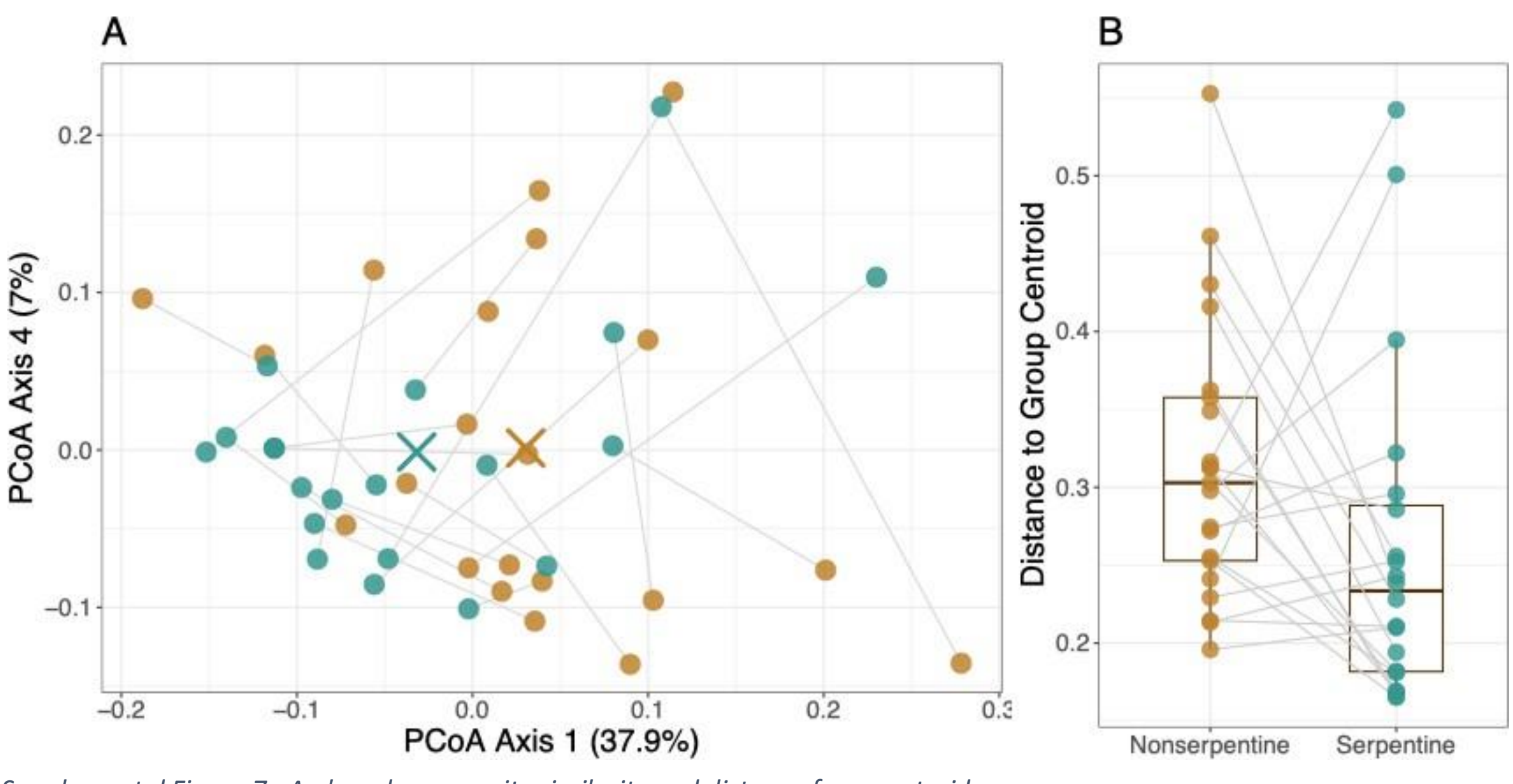

Supplemental Figure 7 - Archaeal community similarity and distance from centroid

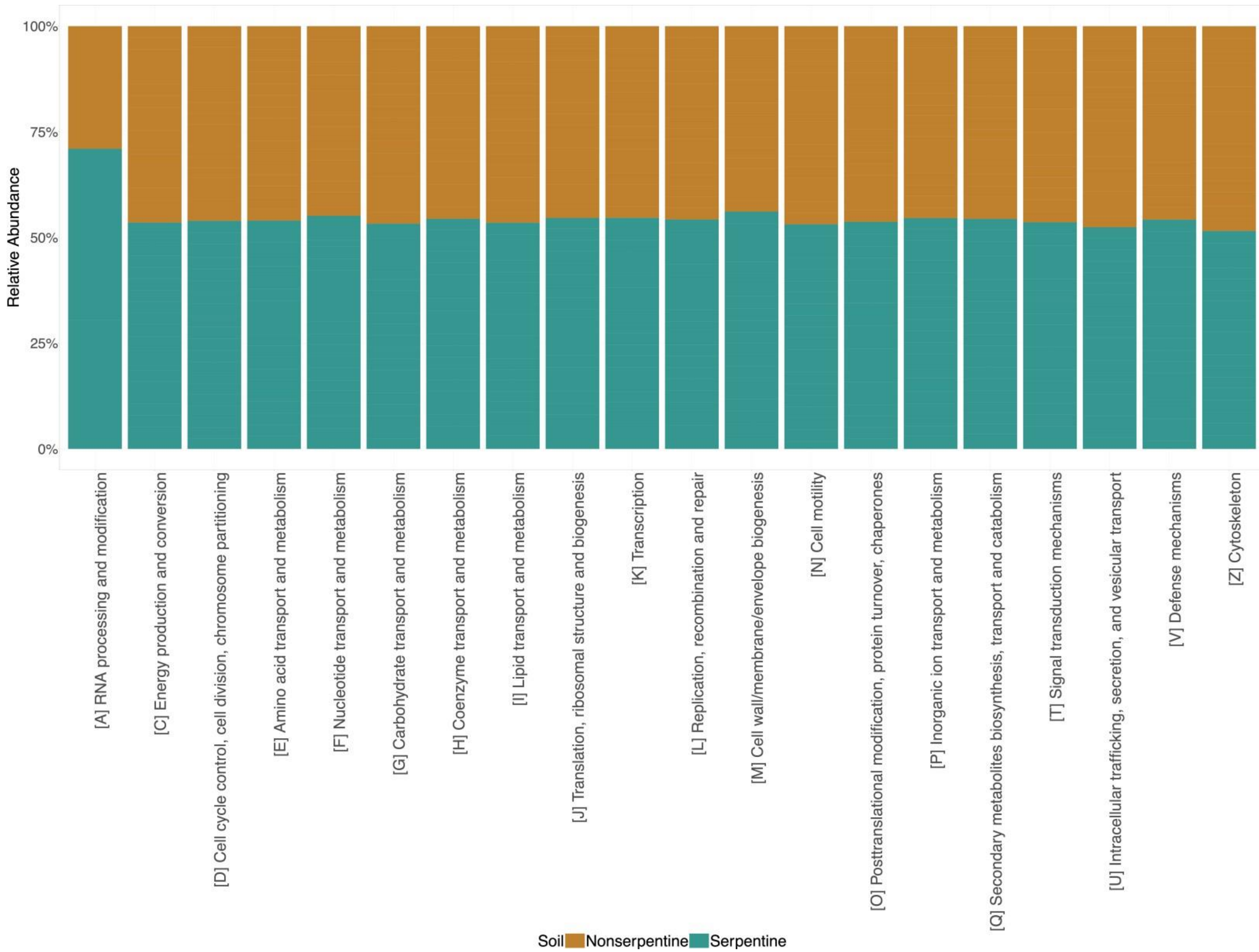

Supplemental Figure 8 - Functional abundances

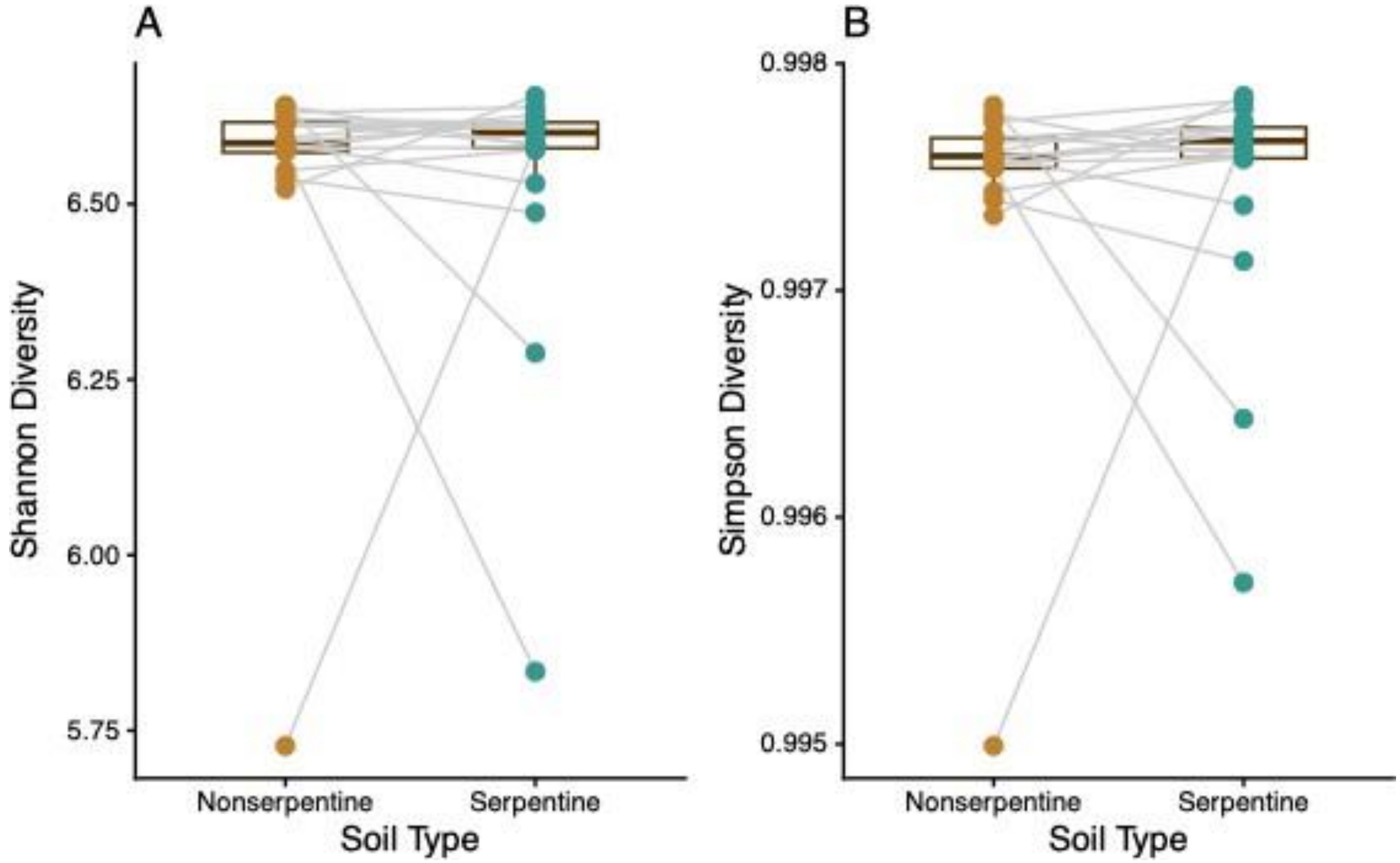

Supplemental Figure 9 - Functional richness
